# Genetically-regulated transcriptomics & copy number variation of proctitis points to altered mitochondrial and DNA repair mechanisms in individuals of European ancestry

**DOI:** 10.1186/s12885-020-07457-1

**Published:** 2020-10-02

**Authors:** Gita A. Pathak, Renato Polimanti, Talisa K. Silzer, Frank R. Wendt, Ranajit Chakraborty, Nicole R. Phillips

**Affiliations:** 1 Department of Microbiology, Immunology & Genetics, Graduate School of Biomedical Sciences, University of North Texas Health Science Center, 3500 Camp Bowie Blvd, Fort Worth, TX 76107 USA; 2 Department of Psychiatry, Yale School of Medicine, Yale University, New Haven, CT USA; 3 Veteran Affairs Connecticut Healthcare System, West Haven, CT USA

**Keywords:** Mitochondria, DNA repair, Proctitis, Radiotherapy

## Abstract

**Background:**

Proctitis is an inflammation of the rectum and may be induced by radiation treatment for cancer. The genetic heritability of developing radiotoxicity and prior role of genetic variants as being associated with side-effects of radiotherapy necessitates further investigation for underlying molecular mechanisms. In this study, we investigated gene expression regulated by genetic variants, and copy number variation in prostate cancer survivors with radiotoxicity.

**Methods:**

We investigated proctitis as a radiotoxic endpoint in prostate cancer patients who received radiotherapy (*n* = 222). We analyzed the copy number variation and genetically regulated gene expression profiles of whole-blood and prostate tissue associated with proctitis. The SNP and copy number data were genotyped on Affymetrix® Genome-wide Human SNP Array 6.0. Following QC measures, the genotypes were used to obtain gene expression by leveraging GTEx, a reference dataset for gene expression association based on genotype and RNA-seq information for prostate (*n* = 132) and whole-blood tissue (*n* = 369).

**Results:**

In prostate tissue, 62 genes were significantly associated with proctitis, and 98 genes in whole-blood tissue. Six genes - *CABLES2, ATP6AP1L, IFIT5, ATRIP, TELO2*, and *PARD6G* were common to both tissues. The copy number analysis identified seven regions associated with proctitis, one of which (*ALG1L2)* was also associated with proctitis based on transcriptomic profiles in the whole-blood tissue. The genes identified via transcriptomics and copy number variation association were further investigated for enriched pathways and gene ontology. Some of the enriched processes were DNA repair, mitochondrial apoptosis regulation, cell-to-cell signaling interaction processes for renal and urological system, and organismal injury.

**Conclusions:**

We report gene expression changes based on genetic polymorphisms. Integrating gene-network information identified these genes to relate to canonical DNA repair genes and processes. This investigation highlights genes involved in DNA repair processes and mitochondrial malfunction possibly via inflammation. Therefore, it is suggested that larger studies will provide more power to infer the extent of underlying genetic contribution for an individual’s susceptibility to developing radiotoxicity.

## Background

Prostate cancer is one of the most prevalent diseases in older men, with 66 years being the average age at the time of cancer diagnosis [1]. According to the SEER cancer statistics of 2019, approximately 3 million men have been previously diagnosed with prostate cancer and are still alive today. This feat can be credited to the advancement in cancer treatment which has contributed to the 5-year relative survival rate of 90% in prostate cancer survivors [2]. Radiation therapy is one of the primary forms of treatment for prostate cancer, delivered as external beam radiotherapy (EBRT) or brachytherapy. While the dose and precision of radiation delivery to the tumor tissue has improved over the years, surrounding normal tissues get irradiated leading to clinical side effects [3]. Proctitis is the inflammation of the rectum, which can result from receiving radiation therapy around the pelvic region such as during prostate cancer treatment [4]. The inflammation of the rectum can either be acute or chronic. Acute proctitis appears within 3 months of receiving radiation therapy, and progression of rectal inflammation after 3 months of completing radiation therapy is identified as chronic proctitis [5]. The development of radiation-induced chronic proctitis affects 5–20% of cancer survivors and is relatively more common [6] than acute proctitis which affects approximately 13% of the cancer population [5]. As of 2016, the total population of cancer survivors in the US was estimated to be approximately 15 million, and by the year 2026 is expected to reach 20 million individuals [7]. Given the prevalence of chronic proctitis affecting cancer individuals (5–20%), we can deduce that approximately 1–4 million cancer survivors experience proctitis from receiving radiotherapy. The goal of radiation therapy in treating cancer is to damage the DNA of cancer tissue by creating double-strand breaks (DSBs). While the cellular system is capable of repairing breaks in the DNA, strands with DSBs are difficult to restore leading to activation of apoptotic signals which ultimately kills cancer cells. Unfortunately, the normal tissue around the targeted region is also affected by DNA damage from radiation, and must rely on DNA repair mechanisms for rehabilitation of cellular functions [8]. A recent twin-study has reported that certain SNPs and their transcriptomic influence are associated with individual radiation sensitivity and a heritability estimate of 66% [9]. Therefore, it is vital to understand genetics underlying molecular mechanisms involved in adverse effects of radiotherapy and individual genetic variations that may induce radiation sensitivity. Genome-wide studies have been conducted to identify gene variants that may contribute towards developing radiotoxic side-effects. These studies have identified genetic variations involved in DNA repair pathways to be associated with overall radiotoxicity [3, 10]. However, the role of altered gene expression [9] from aggregated single nucleotide polymorphisms (SNPs) remains elusive in radiotoxicity phenotypes (e.g., proctitis). Regulatory variants are SNPs within coding regions which contribute towards tissue – specific gene expression alterations leading to variations in the phenotypic spectrum. Estimating the contribution of SNP aggregates to gene expression can be carried out using correlation weights derived from reference datasets which contain both SNP and RNA-seq information as modelled in PrediXcan [11]. One such dataset is the GTEx project, an NIH funded initiative that stores genotype and RNA-seq data of 53 tissues from 620 donors (v7). The majority of the donors in the GTEx dataset are Caucasian, and more than 50% of the donors are over the age of 50 years. These characteristics make GTEx an excellent reference dataset to derive gene expression values from individual level SNP profiles of prostate cancer patients who have received radiation treatment.

Beyond SNPs, genetic discordance from gene dosage and structural effects can be attributed to copy number variation (CNV). CNVs are segments of duplicated DNA that are greater than 1 kb with differences in size between the two copies [12]. In a clinical setting, testing of CNVs is relatively more common than other genetic tests [13] due to the majority of phenotypic changes being associated with these variations in segment size. CNVs associated with radiotoxicity phenotypes (e.g. proctitis) have not been investigated extensively and may prove to be significant contributors to phenotype risk.

We hypothesize that genotype-regulated gene expression profiles and variations in copy number will identify genetic alterations associated with a spectrum of DNA repair functions. Here, we investigate both CNV and tissue specific (prostate and whole-blood) transcriptomic profiles derived from individual-level SNPs that are associated with radiation induced proctitis in prostate cancer patients.

## Methods

The overall methodology is visually summarized in Fig. S1.

### Data access to study subjects

The Gene-PARE was approved by the Institutional Review Board of the Icahn School of Medicine at Mount Sinai and Florida Radiation Oncology Group (Kerns et al. [3, 14]). All patients provided informed consent under the parent study – Gene-PARE, at Icahn School of Medicine, Mount Sinai and Florida Radiation Oncology Group (Kerns et al. [3, 14]). We obtained access to anonymized individual level genotype data from Genetic Predictors of Adverse Radiotherapy Effects (Gene-PARE) (phs000772.v1.p1) via dbGaP’s authorized application, under the approval of North Texas Regional IRB protocol 2016–090. The study described here was performed under the North Texas Regional IRB (formerly the University of North Texas Health Science Center IRB), and was given “EXEMPT” status based on the criteria that our study involved data available from public repository, i.e. dbGaP and does not require approval of receiving informed consent. We analyzed prostate cancer individuals from the discovery set (*N* = 367), which were genotyped for 934,940 SNPs on Affymetrix® Genome-wide Human SNP Array 6.0. The dataset contains phenotypic information on prostate cancer patients who have received radiation treatment either via EBRT or brachytherapy. Out of three radiotoxicity phenotypes – erectile dysfunction, proctitis and urinary morbidity (IPSS/AUASS) – we focused our investigation on proctitis because (1) it is also prevalent in other pelvic region cancers [15] and (2) the data for proctitis was complete for all individuals.

### SNP-QC

We extracted SNP data from the intensity (*.CEL) files using Affymetrix® Genotyping Console using the BIRDSEED v2 algorithm and genotype call rate of 95% and default settings from the array, leaving 905,280 markers and total of 355 individuals. The files were then exported to plink [16] format to perform QC measures as suggested by Anderson et al. [17]. At the individual-level filtering, we removed 5 individuals for either failing heterozygosity or having greater than 9% missing genotypes. At the IBD filter of 0.1875, we removed 29 individuals; further, 120 individuals were removed who were not of European ancestry or failed to cluster with the main patient population based on principal component (PC) analysis of PC1 and PC2 of the genetic data. After SNP-level filtering on SNP missingness, minimum allele frequency and Hardy-Weinberg equilibrium, we had 746,684 SNPs and 222 individuals. The final cohort characteristics after QC were analyzed using Fischer’s exact test for categorical variables and Student’s t-test for continuous variables (Table 1).

**Table 1:** Characteristics of individuals with prostate cancer who received radiotherapy

	Prostate cancer individuals without proctitis; Controls (***N*** = 177)	Prostate cancer individuals with proctitis; Cases (***N*** = 45)	***P***-value
***Mean ± SD***			
**Age**	63.7 ± 7.35	65. 28 ± 7.69	0.25
**Gleason score**	6.45 ± 0.78	6.31 ± 0.72	0.26
***N (%)***			
**Smoking**			
Yes	67 (38%)	14 (31%)	0.48
No	110 (62%)	31 (69%)	
**Diabetes**			
Yes	5 (3%)	3 (7%)	0.21
No	172 (97%)	42 (93%)	
**Hypertension**			
Yes	55 (31%)	10 (22%)	0.28
No	122 (69%)	35 (78%)	
**Treatment**			
EBRT & Brachytherapy	73 (41%)	21 (47%)	0.15
EBRT	0 (0%)	1 (2%)	
Brachytherapy	104 (59%)	23 (51%)	

### Genetically-regulated gene expression and GSEA

Tissue specific gene expression prediction using each individual’s genotype profile was performed using PrediXcan [11]. Integrating expression quantitative trait loci (eQTLs) with genotype increases power to detect gene-based associations with phenotype of interest. The PrediXcan approach determines gene expression attributed to genetic variants, the aggregated effect of local (cis) genetic variants to the gene expression is tested for association with the phenotype. The PrediXcan model, derives weights of SNPs and tissue specific gene expression using lasso regression from GTEx [18] as reference dataset, including variance and covariance patterns based on linkage disequilibrium of cis-SNPs. We obtained model weights for prostate and whole-blood tissues for the GTEx-v7 reference panel, accessible at http://predictdb.org/; filename- GTEx-V7_HapMap-2017-11-29.tar.gz. In the GTEx (v7), there are 132 prostate tissue donors and 369 donors for whole-blood tissue. PrediXcan implements gene expression value prediction, followed by gene-based association. The z-scores identify the direction of expression [19] for each gene and their corresponding *p*-values for association testing. For prostate tissue, a total of 3113 gene and 5954 genes for whole-blood tissue were imputed for their tissue-specific gene expression based on genotype variation. Following association tests, significant genes (identified as *p*-value < 0.05) were investigated further by constructing tissue-specific protein-protein interaction (PPI) networks between query (significant genes) and interacting genes using the DifferentialNet [20] database and NetworkAnalyst3.0 [21]. The network was filtered on betweenness centrality of 4.0 in order to reduce isolated neighboring nodes (each gene is a node). All the genes in the network were subsequently analyzed for gene set enrichment using clusterProfiler [22] for gene ontology and visualized in GOPlot [23].

### Replication of transcriptomic findings with second cohort

We identified the study by Oorschot et al. [24] as an external cohort for replication of our transcriptomic findings. Their study recruited 200 patients who received EBRT for prostate cancer. Prior to receiving treatment, whole blood was drawn, and lymphocytes were cultured, irradiating half of the cells with 2Gy gamma rays and other half left untreated followed by genome-wide microarray analysis. The data were deposited in Gene Expression Omnibus - GSE85570 (https://www.ncbi.nlm.nih.gov/geo/query/acc.cgi?acc=GSE85570). Further details of the study have been described elsewhere [24]. We used BART [25] and NCBI’s GEO2R to analyze differentially expressed genes between untreated and treated with 2 Gy of radiation groups. We tested the significance of genes overlapping our results from prostate and whole-blood tissue to those identified in the replication dataset using GeneOverlap (https://github.com/shenlab-sinai/geneoverlap) which applies Fisher’s exact test testing significant genes for whole blood tissue (*n* = 98), prostate tissue (*n* = 62) and replication cohort (*n* = 2973) against genome (*n* = 21,196) .

### CNV association and GSEA

The intensity (*.CEL) files of 222 individuals from the above QC protocol were extracted for copy number analysis using Affymetrix® Genotyping Console. Copy number segments were filtered to regions (minimum genomic size of 2 kbps) with 10 marker per segment [26]. The copy number data was exported as tab-delimited file for copy number association in CNVRuler [27]. CNV regions were significantly associated at FDR *p*-value < 0.05. The significant CNV regions were visualized using Phenogram [28] then mapped to genes using UCSC browser for GRCh37/hg19 assembly (https://genome.ucsc.edu/). The genes within CNV regions were analyzed for functional and diseases processes and visualized using Ingenuity Pathway Analysis (QIAGEN Inc., https://www.qiagenbioinformatics.com/products/ingenuity-pathway-analysis).

## Results

### Genes identified in prostate tissue

**Fig. 1:**
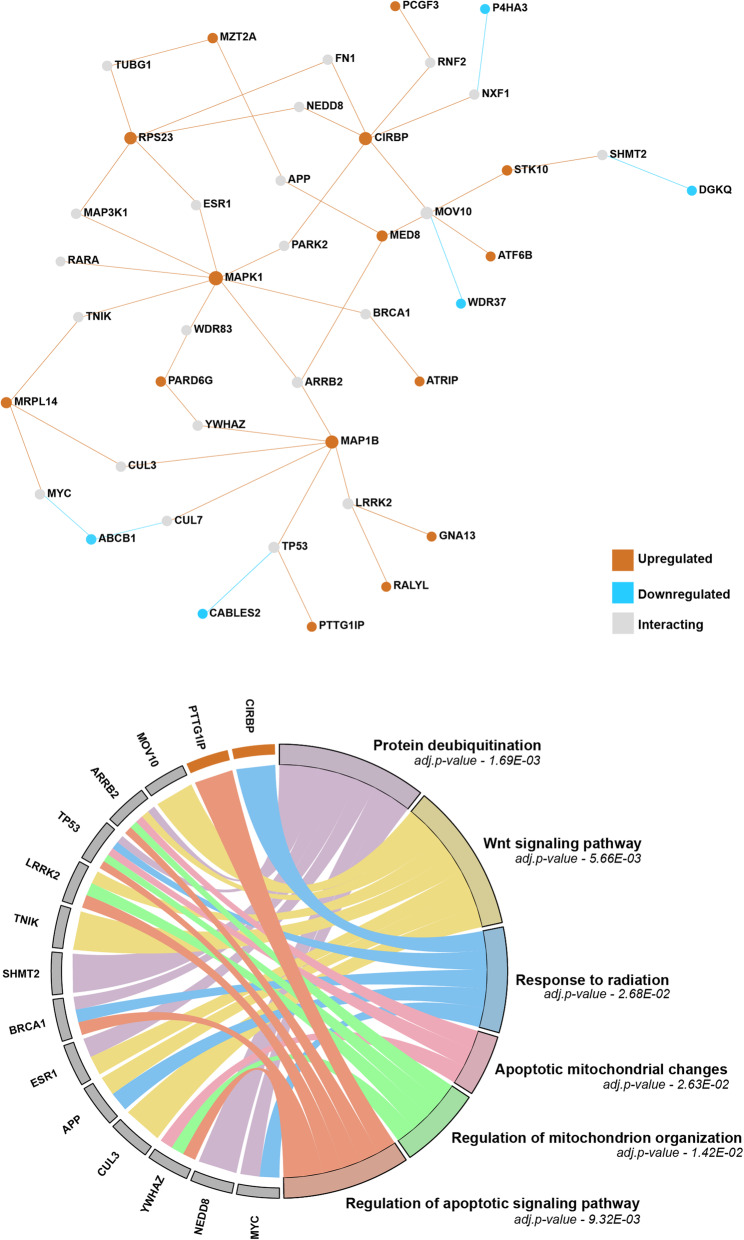
Gene Expression of prostate tissue. **a**. Genes that are upregulated are shown as orange nodes in the network and downregulated genes are shown as blue nodes. The grey nodes are interacting nodes derived from prostate tissue specific PPI-information. **b**. The chord plot summarizes enriched gene ontology pathways of the genes from the network shown in the top panel. The FDR *p*-value of each pathway is shown under the name of the GO category. See Table S2 in Supplementary file for more details

In the association analysis between prostate cancer individuals who developed proctitis (cases) and who didn’t develop proctitis (controls), we found a total of 62 differentially expressed genes to be significantly associated. Based on z-score direction, 28 genes were downregulated, and 34 genes were upregulated in the prostate tissue (Table S1). We mapped the genes to tissue-specific protein-protein interaction (PPI) network to understand combined functional relationship of differentially expressed genes. Integrating PPI information with identified genes, we found an additional 22 genes which interact with 20 genes associated with proctitis based on genetically regulated expression of prostate tissue. Analyzing all the genes in the network for enriched gene ontology of biological processes, molecular functions and cellular components (Fig. 1) highlighted protein deubiquitination (*ARRB2, TP53, SHMT2, BRCA1, ESR1, NEDD8, MYC*), wnt signaling (*MOV10, ARRB2, LRRK2, TNIK, ESR1, APP, CUL3*), regulation of apoptosis signaling (*ARRB2, TP53, LRRK2, BRCA1, YWHAZ, PTTG1IP*), response to radiation (*CIRBP, TP53, BRCA1, APP, MYC*) and mitochondrial organization & apoptotic mitochondrial changes (*ARRB2, TP53, LRRK2, YWHAZ*) (Table S2). Specifically, gene ontology categories that included genes identified via SNP-based gene expression were ‘response to radiation’ and ‘regulation of apoptotic signaling pathway’.

### Genes identified in whole blood tissue

**Fig. 2:**
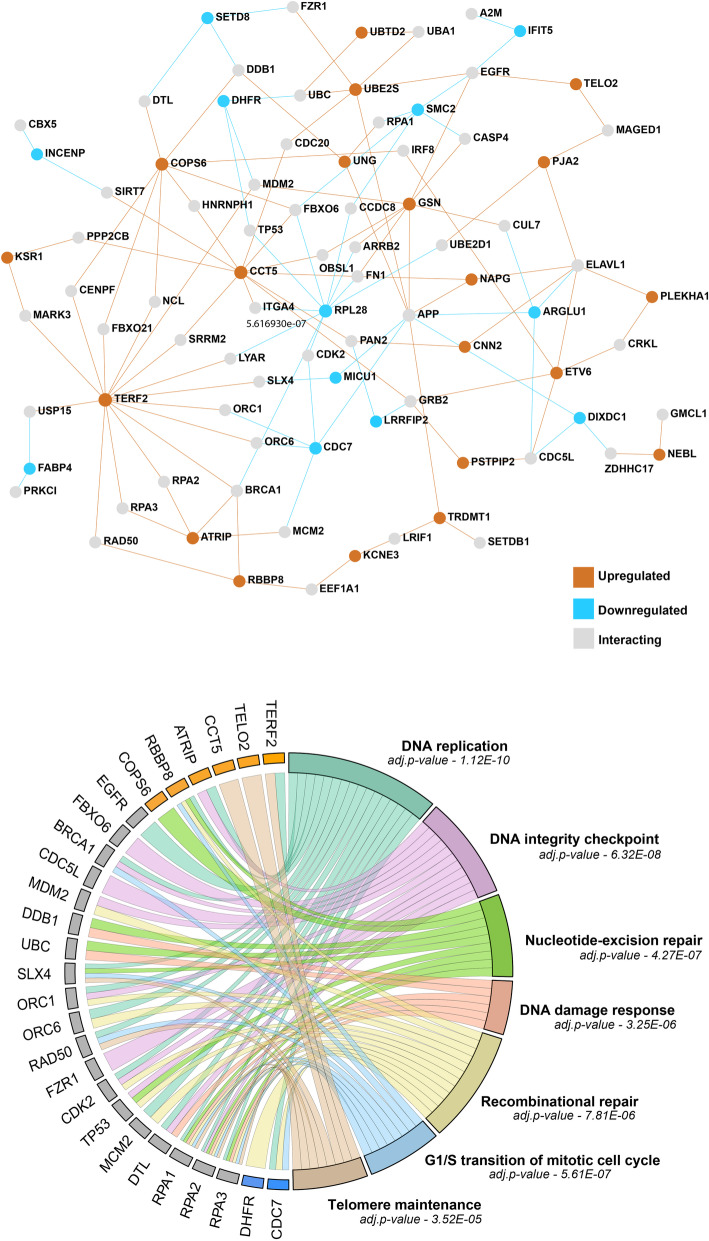
Gene expression for whole blood tissue. **a**. Genes that are upregulated are shown as orange nodes in the network and downregulated genes are shown as blue nodes. The grey nodes are interacting nodes derived from whole-blood specific PPI-information. **b**. The chord plot summarizes enriched gene ontology pathways of the genes from the network shown in the top panel. The FDR p-value of each pathway is shown under the name of the GO category. See Table S4 in Supplementary file for more details

We found a total of 98 genes to be associated with proctitis in whole blood tissue. 49 genes were upregulated, and 49 genes were downregulated (Table S3). Integrating PPI network information with the significant genes, found an additional 51 intermediate genes that interact with identified genes based on genetically regulated gene expression from whole blood. Investigating all the genes in the network for over-represented biological processes, highlighted DNA repair processes (Fig. 2) such as DNA replication (*TERF2, EGFR, CDC7, BRCA1, ATRIP, RBBP8, SLX4, ORC1, ORC6, RAD50, CDK2, MCM2, DTL, RPA1, RPA2, RPA3*), DNA integrity checkpoint (*FBXO6, BRCA1, CDC5L, ATRIP, MDM2, ORC1, FZR1, CDK2, TP53, DTL, RPA2*), nucleotide excision repair (*COPS6, RBBP8, DDB1, UBC, SLX4, TP53, RPA1, RPA2, RPA3*), recombinational repair (*CDC7, BRCA1, RBBP8, SLX4, RAD50, RPA1, RPA2, RPA3*), detection of DNA damage and response (*DDB1,UBC,DTL,RPA1,RPA2,RPA3*) and telomeric maintenance (*TERF2, CCT5, SLX4, RAD50, TELO2, RPA1, RPA2, RPA3*) (Table S4). Thus, integrating PPI information allows for the inference of gene-network as the gene expression changes attributed to genetic variants is limited in identifying all gene members of DNA damage and its related networks.

### Replication dataset for transcriptomic findings

**Fig. 3:**
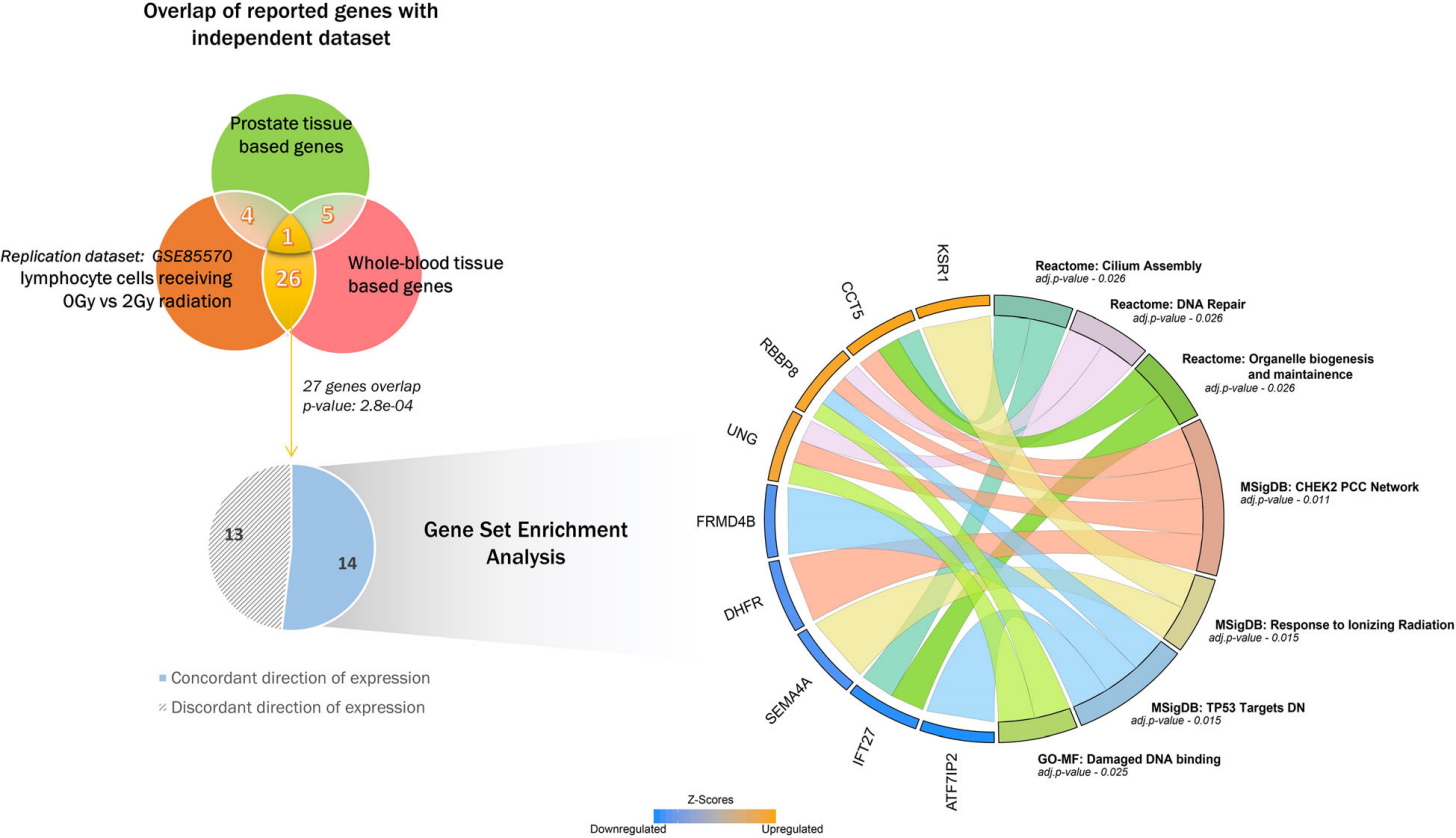
Genes overlapping with external cohort. In the Venn diagram, we highlight the number of significant genes overlapping between our transcriptomic associations of proctitis (radiotoxicity phenotype) for prostate and whole-blood tissue and an external dataset with similar phenotype assessing differential expression between groups receiving untreated vs 2Gy of radiation. The overlap of 27 genes between whole-blood and external dataset is significant. 14/27 genes had same direction of expression in both datasets and were further analyzed for GSEA. The chord plot shows the representative Database: Name of function category on the right side of the circle and genes present in these categories are on the left connected with strings. The up/downregulation of genes is reported via their z-scores. See Supplementary file for details

In order to replicate our genetically-regulated gene expression genes associated with radiation toxicity-proctitis, we identified the study of van Oorschot et al., whose data were deposited in gene expression omnibus (GEO) [24]. Their study isolated and cultured lymphocytes from individuals who received radiation treatment for their prostate cancer and were assessed for radiotoxicity for a period of 2 years. In their study design they irradiated lymphocytes (collected prior to radiation treatment) with untreated and 2Gy of gamma ray, followed by microarray analysis. We analyzed differential expression of genes between the two irradiated groups. A total of 27 genes overlapped between significant genes identified in replication cohort and significant genes in whole blood tissue (overlapping p-value: 0.00028), and 5 genes overlapped with significant genes in prostate tissue. We further investigated the 27 genes that significantly overlapped with the external cohort for direction of gene expression. We found 14 genes to have concordant direction of expression between both results whereas the remaining 13 genes were discordant. The GSEA analysis of these 14 genes was enriched for Cilium Assembly, DNA repair, Organelle biogenesis and maintenance, CHEK2 PCC Network, Response to Ionizing radiation, TP53 Targets and Damaged DNA binding (Fig. 3; Supplementary file – Table S5).

### CNV association and GSEA of mapped genes

**Fig. 4:**
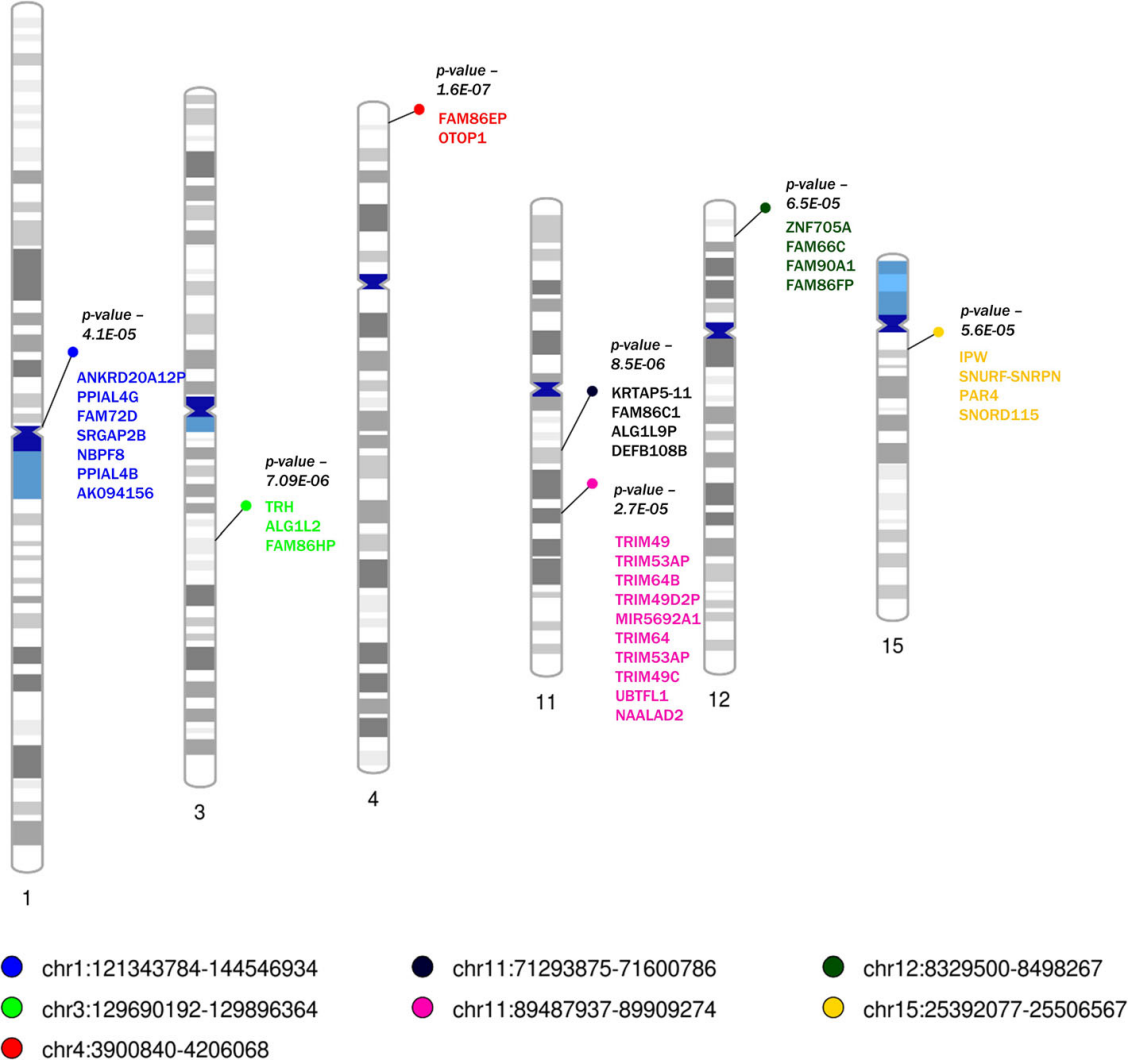
Copy Number Variation Analysis. Significant CNV regions are labelled on the ideogram with their corresponding *p*-values. Each region is highlighted with circles and its legend is shown at the bottom. The coding genes within each CNV region is shown in the same color as the regions color from the legend. For full list of annotated molecules, please see supplementary files

**Fig. 5:**
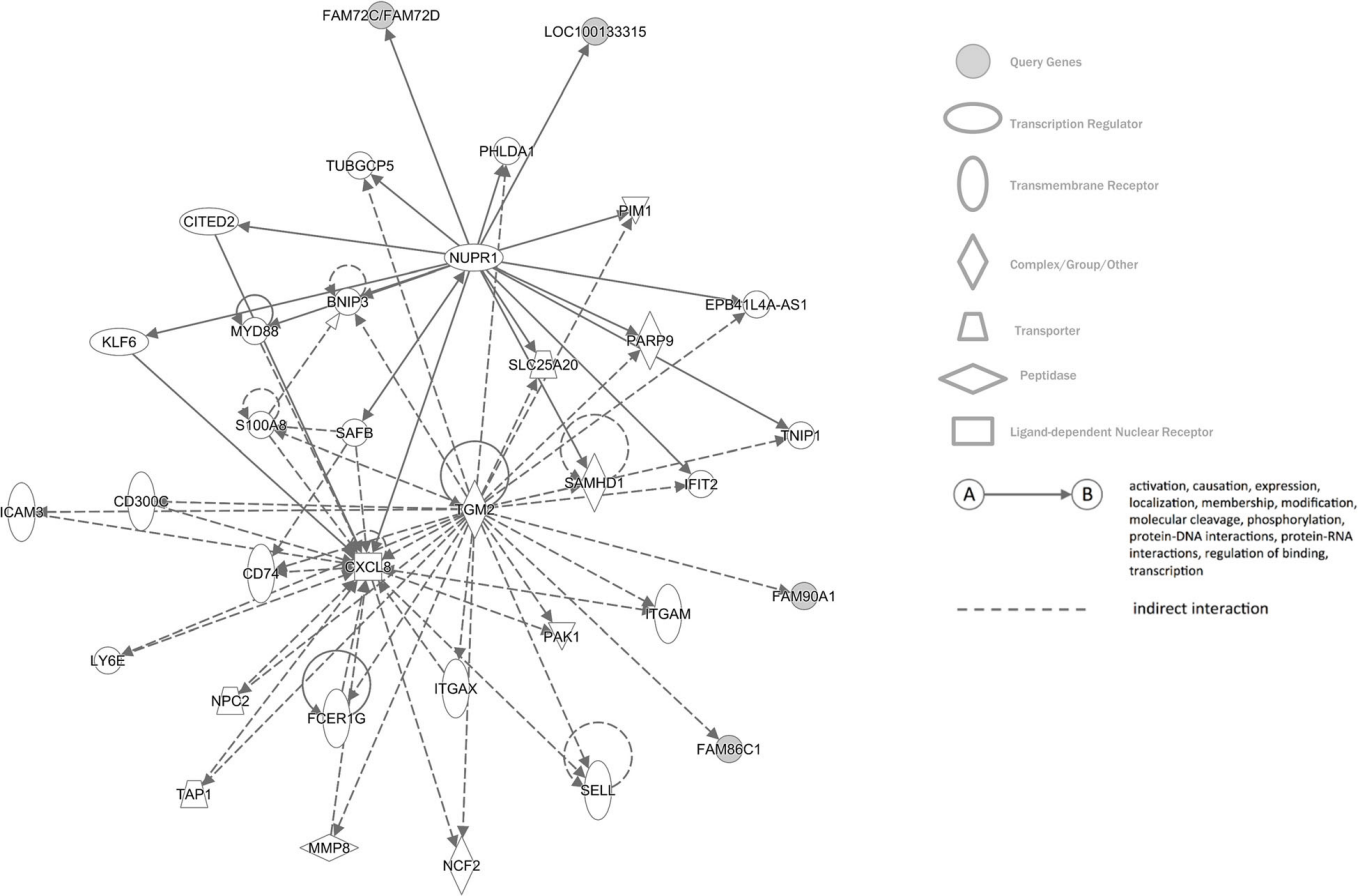
Network identified for genes identified in significant CNV regions. The network was generated using IPA®, the grey filled circles are query genes from the identified CNV regions. Two genes – NUPR1 and TGM2 were found to interact with the query genes

We found 7 CNV regions associated with proctitis on chromosomes 1, 3, 4, 11, 12 and 15 (Fig. 4; Table S7). We identified genes within CNV regions using the UCSC browser (hg19) (Table S8). Interestingly, out of the two regions on chromosome 11 that were significant, we observed a high number of TRIM family genes (chr11:89487937–89,909,274 bps). The mapped genes from copy number regions were investigated for gene interactions using biobase knowledge of Ingenuity Pathway Analysis®. The pathway with the highest number of query genes (Fig. 5) was further analyzed for enriched disease and functional categories (Table S9). Cell-to-cell signaling interaction processes for renal and urological system, connective tissue development and function, and organismal injury were significantly associated processes, and their functional categorization included synthesis, proliferation, apoptosis and transmembrane transport. It is interesting to note, that most of these processes were dominated by *TRH* and *TRIM*-family genes. Furthermore, we also observed that the *ALG1L2* gene, which was one of the significantly downregulated genes in whole-blood tissue, was also mapped to significant CNV region on chr3:129690192–129,896,364 bps which observes both gain and loss of copy, referred to as mixed regions.

## Discussion

Genetic susceptibility towards developing radiotoxic phenotypes is an upcoming research interest of significant clinical impact to improve the quality of life of cancer survivors [8]. Previously, GWAS studies have been conducted to identify genetic loci associated with overall toxicity, decreased urinary stream, and erectile dysfunction [10, 29] in prostate cancer individuals who received radiotherapy [30]. While these findings have shed much-needed light on SNP loci associated with susceptibility towards radiotoxicity, cumulative effects of exonic SNPs on gene expression and other genetic alterations such as copy number differences have not been previously studied. Here, we integrated genotyping data to identify genetic risk associated with proctitis by (1) employing genetic variant–derived gene expression of both prostate and whole-blood tissue, and (2) identifying associated genomic CNVs. The transcriptomic analyses points to several novel genes that play role in DNA-repair processes. In addition, we identified variable copy number regions that had multiple members of TRIM-family genes to be associated with proctitis. Along with novel genes identified through the analysis, the incorporation of PPI map reveals convergence of the implicated gene sets on known DNA-repair, mitochondrial, and telomeric regulation processes, highlighting their involvement with radiotoxic phenotypes (e.g. proctitis).

Six genes from both prostate and whole-blood tissue were associated with proctitis. *CABLES2, ATP6AP1L and IFIT5* were under expressed, and *ATRIP* and *TELO2* were upregulated in both tissues, however *PARD6G* was over expressed in prostate tissue and under expressed in whole blood tissue. *CABLES2* (Cdk5 And Abl Enzyme Substrate 2), which is involved in regulation of the cell cycle, was also reported to be under expressed in lymphocytes of occupational workers who were exposed to ionizing radiation [31]. *ATP6AP1L* (ATPase H+ Transporting Accessory Protein 1 Like) is critical for proton transportation and ATP synthase activity in the mitochondria; it has a paralog, ATP6AP1, which is involved in secretory granules and regulating neuroendocrine responses [32]. *IFIT5* (IFN-induced protein with tetratricopeptide repeats) has been reported to act as an enhancer in immune responses, with partial containment in mitochondria [33]. A recent study has reported that elevated *IFIT5* gene expression was correlated with interferon-γ levels in prostate cancer individuals after radiation, and demonstrated that IFN-γ stimulated epithelial-to-mesenchymal transition through the activation of JAK-STAT pathway [34]. *ATRIP* [*TREX1*] is an ATR interacting protein that is a DNA exonuclease [35] that is known to initiate DNA repair pathways [36]. *ATRIP* has also been reported to provide telomere protection by recruiting *ATM* – a key player in regulating cellular damage responses— to telomeric and DNA damage sites unaided by *ATR* kinase activity [37]. Additionally, *ATR* responds to UV damage via the downregulation of Pin1 demonstrating anti-apoptotic activity in mitochondria [38]. *ATRIP* [*TREX1*] has been shown to be upregulated in radiation-induced immunogenicity of tumor cells [39] by degrading cytosolic dsDNA and transferring cancer cells to dendritic cells under the stimulation of interferon-type1 [40]. In addition to *ATRIP*, *TELO2* [*CLK2*] also interacts with ATM to stimulate cell cycle arrest in response to radiation induced double strand breaks [41] via AKT activation [42]. High *TELO2* expression activity has been identified to be correlated with cell protection when exposed to high radiation doses [42]; conversely, *TELO2* overexpression can trigger inflammation by influencing PIKKs (via *mTORC1* binding [43]) while responding to DNA damage [44]. *PARD6G* was found to have opposite direction of expression in prostate and whole-blood tissue, which could be attributed to tissue specific differences. Hypermethylation and downregulation of PARD6G was concluded to be involved in DNA repair mechanisms [45] in bisphenol A (BPA, a xenoestrogen) exposed human-derived breast cancer epithelial cells.

Assessment of copy number variation associated with radiation toxicity phenotypes can help identify genetic alterations that may lead to functional changes in gene expression, and thus, phenotypic variation. While CNVs have recently gathered interest in cancer diagnosis and treatment, their importance in studying radiation toxicity phenotypes remains understudied. Here, analysis of copy number data identified 7 regions to be associated with proctitis. The *ALG1L2* mapped to the significant CNV region on chr3:129690192–129,896,364 (along with *TRH* and *FAM86HP)* was also found to be associated with proctitis in the transcriptomic analysis of whole-blood tissue, while the other two genes (*TRH* & *FAM86HP*) were not predicted in either of the two tissues. Based on gene ontology annotations, *ALG1L2* functions as a mannosyl transferase in protein glycosylation [46]. Another gene within chromosome 3 CNV region is *TRH* (Thyrotropin releasing hormone); its function includes carbohydrate and amino acid metabolism, and it has been involved in endocrine system disorders, metabolic disease, and organismal injury (Table S7). *TRH* has been shown to mobilize calcium from endoplasmic reticulum and mitochondria [47], and plays role in mitochondrial endoxidation via mitochondrial complex I and IV enzyme activity in skin samples [48], which is aligned with *TRH*’s known involvement in hair and skin development (as indicated in our IPA results, Table S7). Variants in *FAM86HP* (Family With Sequence Similarity 86 Member H, Pseudogene) have been reported to be associated with BMI-adjusted waist-hip ratio [49]. In the pathway analysis (Fig. 5), we observed that *FAM86HP* has an indirect interaction with *TGM2* (Transglutaminase 2), a stress-response gene [50] involved in mRNA metabolism [51] which has been reported to be upregulated during inflammation [50]. It is interesting to note that *TGM2* was over expressed in individuals receiving chemo & radiation therapy, suggesting its involvement in sensitivity to radiation [52, 53]. Both, *TGM2* and *NUPR1* (Fig. 5) have been reported to be implicated in inflammation driven primarily via the JAK/Stat and IL-17A signaling pathway [54]. In the pathway we observe, *NUPR1* has direct interaction with *FAM72C/FAM27D* and LOC100133315, both of which were identified from the copy number variation association. *NUPR1* is known to repair double strand DNA breaks [55] and regulate cell cycle progression [56] from damage induced by gamma irradiation [55]. The under expression of *NUPR1* has been reported to result in increased ROS production thus creating a deficit in mitochondrial membrane potential. This alteration in OXPHOS activity has been associated with ER stress and triggers programmed necrosis in cancer cells [57]. During angiogenesis in cancer cells, *NUPR1* was reported to be upregulated in association with triiodothyronine thyroid hormone receptor [58], which is regulated with TRH - Thyrotropin releasing hormone [59]. It is interesting to note that *NUPR1* has been reported to play different roles before cancer development, during cancer progression and in response to cancer treatment.

Normal tissues receive varying amounts of radiation depending on their proximity to the tumor tissue, thus exhibiting a spectrum of toxicity effects. Genome-wide and molecular studies have shown that alterations in the DNA-damage response (DDR) from ATM (ataxia-telangiectasia mutation) mutations influence intraindividual variations to radiation toxicity [60]. The findings in this study observe that genes such as *ATRIP, NUPR1, TELO2*, and *TRH* have multiple roles in damage detection and response mechanism. The genes and their PPI networks highlight their involvement in cell cycle arrest upon detection of DNA damage, affecting DNA replication and repair. During course of DDR, ROS from radiation seems to promote mitochondrial-induced apoptosis [61]. Further, a constant but varying amount of inflammatory responses due to tissue injury (normal and tumor) appears to trigger ROS–induced mitochondrial oxidation [62], which exacerbates local inflammation in nearby tissues and propagates this DNA repair-inflammation stress cycle. Our findings report several novel genes that have been observed to be associated with known *BRCA1-ATM-RAD50* damage response complex [63] which are activated in response to radiation, thus extending our understanding of these new players and their multifactorial roles associated with proctitis.

Our study has several limitations. The sample size is small, and our findings should be replicated and functionally validated in future studies using an animal model for targeted effects of radiotoxicity. Our study concentrated on prostate cancer survivors of Caucasian ethnicity, hence, to understand if similar or different genes are associated with proctitis developed during treatment of other cancers, it is imperative that our methods be applied to other races/ethnicities and other cancers. In the CNV regions, we mapped several noncoding regions, including lcRNA and snRNAs that would require fine mapping to further understand their involvement with proctitis. Unfortunately, there are only a handful of studies that investigate genetics underlying radiotoxicity phenotypes.

There are also many strengths to our study. Leveraging SNPs for reference transcriptomic data and copy number association, we identified several novel genes associated with proctitis – an inflammation of the rectum resulting from radiation therapy received for prostate cancer. The integration of tissue - specific PPI network aided in understanding the biological interactions between known and reported genes. We were able to replicate 27 of our genes to be significantly representative in an external cohort of prostate cancer diagnosis receiving radiation treatment. Analysis of copy number variation identified several genes in the reported regions and their pathways analysis highlighted two primary genes that have distinctive roles before cancer development and in response to cancer treatment.

## Conclusions

In conclusion, this investigation highlights genes primarily involved in DNA repair processes and mitochondrial malfunction threaded via inflammation. The field of radiogenomics –work investigating the role of genetics in developing radiation toxicity—calls for investigation of genetic risk that can help inform dose management of radiation treatment and toxicity monitoring during treatment [8]. We anticipate that understanding genetic data from both CNV and SNPs would contribute towards optimization of radiation treatment on an individual basis. Similar studies in the future would play strong role in early clinical interventions or periodic checkups for individuals who have high expression of DNA damage activity and alterations in copy number within specific regions.

## Supplementary information

**Additional file 1.**

## Data Availability

All results generated or analyzed during this study are included in this article (and its Supplementary Information files). Original genotype data can be accessed via dbGaP. The model weights for PrediXcan for GTEx genotype-gene expression associations can be accessed from predictdb.org, and external cohort can be accessed on Gene Expression Omnibus.

## Abbreviations

SNP: Single nucleotide polymorphism
CNV: Copy Number Variation
PPI: Protein-Protein interaction
lcRNA: long coding RNA
GSEA: Gene Set Enrichment Analysis
IPA: Ingenuity Pathway Analysis
EBRT: External beam radiotherapy
