# Supplementary material for: Genetically-regulated transcriptomics & copy number variation of proctitis points to altered mitochondrial and DNA repair mechanisms in individuals of European ancestry
Source: BMC Cancer. 2020 Oct 2;20:954. doi: 10.1186/s12885-020-07457-1 (PMC7530964; doi:10.1186/s12885-020-07457-1)

SUPPLEMENTARY FILE

Genetically-regulated transcriptomics & copy number variation of proctitis points to altered mitochondrial and DNA repair mechanisms in individuals of European ancestry

Gita A Pathak[1], Renato Polimanti[2,3], Talisa K Silzer[1], Frank R Wendt[2,3], Ranajit Chakraborty[1‡], Nicole R Phillips[1] ✉

[1] Department of Microbiology, Immunology & Genetics, Graduate School of Biomedical Sciences, University of North Texas Health Science Center, Fort Worth, Texas, 76107, USA

[2] Department of Psychiatry, Yale School of Medicine, Yale University, New Haven, CT

[3] Veteran Affairs Connecticut Healthcare System, West Haven, CT

[‡] Deceased

✉ To whom correspondence should be addressed

Nicole R. Phillips
Email:nicole.phillips@unthsc.edu

Table of Contents

Tables

Table S1: Significantly associated genes with proctitis in prostate tissue 3
Table S2: Gene set enrichment for genes identified in the prostate tissue network in Fig.1 5
Table S3: Significant genes associated with proctitis in whole blood tissue.......... 5
Table S4: Gene set enrichment for genes identified in the whole blood tissue network in Fig.2 9
Table S5: Gene set enrichment analysis of overlapping genes with an external cohort (Fig 3) 10
Table S6: Comparison of gene expression direction for the 27 overlapping genes 10
Table S7: Significant copy number variation associated with proctitis............... 11
Table S8: Gene annotation of significant CNV regions from UCSC Browser (GRCh37/hg19) 12
Table S9: Significant functions identified for the network constructed in IPA® (Fig.4) of genes in CNV regions 18

Figures

Figure S1: Visual summary of methodology.. 19

Table S1: Significantly associated genes with proctitis in prostate tissue

Gene (Ensemble ID)	Z-score	P-value	Gene Symbol	Gene symbol definition
Downregulated genes based on Z-scores				
ENSG00000149679.7	-3.07	2.15E-03	CABLES2	Cdk5 and Abl enzyme substrate 2 [Source:HGNC Symbol;Acc:16143]
ENSG00000109794.9	-3.04	2.40E-03	FAM149A	family with sequence similarity 149, member A [Source:HGNC Symbol;Acc:24527]
ENSG00000205464.7	-3.01	2.59E-03	ATP6AP1L	ATPase, H+ transporting, lysosomal accessory protein 1-like [Source:HGNC Symbol;Acc:28091]
ENSG00000152778.7	-2.99	2.79E-03	IFIT5	interferon-induced protein with tetratricopeptide repeats 5 [Source:HGNC Symbol;Acc:13328]
ENSG00000270614.1	-2.96	3.03E-03	CTC-325H20.7	NA
ENSG00000235192.1	-2.92	3.50E-03	AC009495.2	NA
ENSG00000230847.4	-2.89	3.79E-03	RP11-195E2.1	NA
ENSG00000225864.1	-2.88	3.97E-03	HCG4P11	HLA complex group 4 pseudogene 11 [Source:HGNC Symbol;Acc:22930]
ENSG00000047056.10	-2.84	4.46E-03	WDR37	WD repeat domain 37 [Source:HGNC Symbol;Acc:31406]
ENSG00000149380.7	-2.79	5.28E-03	P4HA3	prolyl 4-hydroxylase, alpha polypeptide III [Source:HGNC Symbol;Acc:30135]
ENSG00000254974.1	-2.78	5.38E-03	RP11-702H23.2	NA
ENSG00000145214.9	-2.76	5.77E-03	DGKQ	diacylglycerol kinase, theta 110kDa [Source:HGNC Symbol;Acc:2856]
ENSG00000196683.6	-2.75	5.90E-03	TOMM7	translocase of outer mitochondrial membrane 7 homolog (yeast) [Source:HGNC Symbol;Acc:21648]
ENSG00000177144.5	-2.74	6.09E-03	NUDT4P1	nudix (nucleoside diphosphate linked moiety X)-type motif 4 pseudogene 1 [Source:HGNC Symbol;Acc:18012]
ENSG00000234882.1	-2.72	6.50E-03	EIF3EP1	eukaryotic translation initiation factor 3, subunit E pseudogene 1 [Source:HGNC Symbol;Acc:6102]
ENSG00000255987.1	-2.70	7.03E-03	RP11-1094M14.4	NA
ENSG00000136367.12	-2.67	7.66E-03	ZFHX2	zinc finger homeobox 2 [Source:HGNC Symbol;Acc:20152]
ENSG00000167971.14	-2.66	7.76E-03	CASKIN1	CASK interacting protein 1 [Source:HGNC Symbol;Acc:20879]
ENSG00000185482.3	-2.55	1.06E-02	STAC3	SH3 and cysteine rich domain 3 [Source:HGNC Symbol;Acc:28423]
ENSG00000143630.5	-2.52	1.18E-02	HCN3	hyperpolarization activated cyclic nucleotide-gated potassium channel 3 [Source:HGNC Symbol;Acc:19183]
ENSG00000175749.11	-2.47	1.37E-02	EIF3KP1	eukaryotic translation initiation factor 3, subunit K pseudogene 1 [Source:HGNC Symbol;Acc:44016]
ENSG00000188687.11	-2.46	1.38E-02	SLC4A5	solute carrier family 4, sodium bicarbonate cotransporter, member 5 [Source:HGNC Symbol;Acc:18168]
ENSG00000165055.11	-2.45	1.43E-02	METTL2B	methyltransferase like 2B [Source:HGNC Symbol;Acc:18272]
ENSG00000105767.2	-2.43	1.51E-02	CADM4	cell adhesion molecule 4 [Source:HGNC Symbol;Acc:30825]
ENSG00000085563.10	-2.42	1.55E-02	ABCB1	ATP-binding cassette, sub-family B (MDR/TAP), member 1 [Source:HGNC Symbol;Acc:40]
ENSG00000174238.10	-2.38	1.73E-02	PITPNA	phosphatidylinositol transfer protein, alpha [Source:HGNC Symbol;Acc:9001]
ENSG00000245556.2	-2.38	1.75E-02	CTD-2037K23.2	NA

ENSG00000101751.6	-2.34	1.95E-02	POLI	polymerase (DNA directed) iota [Source:HGNC Symbol;Acc:9182]
Upregulated genes based on Z-scores				
ENSG00000168993.10	2.33	1.98E-02	CPLX1	complexin 1 [Source:HGNC Symbol;Acc:2309]
ENSG00000256682.1	2.34	1.95E-02	TAS2R12	NA
ENSG00000205809.5	2.34	1.93E-02	KLRC2	killer cell lectin-like receptor subfamily C, member 2 [Source:HGNC Symbol;Acc:6375]
ENSG00000206127.6	2.35	1.90E-02	GOLGA8O	golgin A8 family, member O [Source:HGNC Symbol;Acc:44406]
ENSG00000204520.8	2.36	1.83E-02	MICA	MHC class I polypeptide-related sequence A [Source:HGNC Symbol;Acc:7090]
ENSG00000064886.9	2.37	1.76E-02	CHI3L2	chitinase 3-like 2 [Source:HGNC Symbol;Acc:1933]
ENSG00000178184.11	2.40	1.66E-02	PARD6G	par-6 partitioning defective 6 homolog gamma (C. elegans) [Source:HGNC Symbol;Acc:16076]
ENSG00000259865.1	2.41	1.61E-02	RP11-488L18.10	NA
ENSG00000069493.10	2.42	1.55E-02	CLEC2D	C-type lectin domain family 2, member D [Source:HGNC Symbol;Acc:14351]
ENSG00000100439.6	2.44	1.48E-02	ABHD4	abhydrolase domain containing 4 [Source:HGNC Symbol;Acc:20154]
ENSG00000100030.10	2.45	1.44E-02	MAPK1	mitogen-activated protein kinase 1 [Source:HGNC Symbol;Acc:6871]
ENSG00000104228.8	2.49	1.28E-02	TRIM35	tripartite motif containing 35 [Source:HGNC Symbol;Acc:16285]
ENSG00000260911.1	2.49	1.28E-02	RP11-196G11.2	NA
ENSG00000173272.9	2.50	1.25E-02	MZT2A	mitotic spindle organizing protein 2A [Source:HGNC Symbol;Acc:33187]
ENSG00000185619.13	2.52	1.17E-02	PCGF3	polycomb group ring finger 3 [Source:HGNC Symbol;Acc:10066]
ENSG00000183255.7	2.53	1.14E-02	PTTG1IP	pituitary tumor-transforming 1 interacting protein [Source:HGNC Symbol;Acc:13524]
ENSG00000214376.5	2.55	1.08E-02	VSTM5	V-set and transmembrane domain containing 5 [Source:HGNC Symbol;Acc:34443]
ENSG00000184672.7	2.55	1.07E-02	RALYL	RALY RNA binding protein-like [Source:HGNC Symbol;Acc:27036]
ENSG00000213676.6	2.57	1.02E-02	ATF6B	activating transcription factor 6 beta [Source:HGNC Symbol;Acc:2349]
ENSG00000164053.13	2.60	9.46E-03	ATRIP	ATR interacting protein [Source:HGNC Symbol;Acc:33499]
ENSG00000240494.2	2.62	8.73E-03	RPS12P28	ribosomal protein S12 pseudogene 28 [Source:HGNC Symbol;Acc:36972]
ENSG00000145495.10	2.64	8.39E-03	MARCH6	membrane-associated ring finger (C3HC4) 6, E3 ubiquitin protein ligase [Source:HGNC Symbol;Acc:30550]
ENSG00000131711.10	2.68	7.31E-03	MAP1B	microtubule-associated protein 1B [Source:HGNC Symbol;Acc:6836]
ENSG00000113430.5	2.70	6.93E-03	IRX4	iroquois homeobox 4 [Source:HGNC Symbol;Acc:6129]
ENSG00000159479.12	2.71	6.77E-03	MED8	mediator complex subunit 8 [Source:HGNC Symbol;Acc:19971]
ENSG00000123545.5	2.71	6.67E-03	NDUFAF4	NADH dehydrogenase (ubiquinone) complex I, assembly factor 4 [Source:HGNC Symbol;Acc:21034]
ENSG00000180992.5	2.84	4.57E-03	MRPL14	mitochondrial ribosomal protein L14 [Source:HGNC Symbol;Acc:14279]
ENSG00000186468.8	2.87	4.07E-03	RPS23	ribosomal protein S23 [Source:HGNC Symbol;Acc:10410]
ENSG00000099622.9	2.89	3.91E-03	CIRBP	cold inducible RNA binding protein [Source:HGNC Symbol;Acc:1982]

ENSG00000244753.2	2.89	3.79E-03	RPL15P21	ribosomal protein L15 pseudogene 21 [Source:HGNC Symbol;Acc:36190]
ENSG00000120063.5	3.04	2.39E-03	GNA13	guanine nucleotide binding protein (G protein), alpha 13 [Source:HGNC Symbol;Acc:4381]
ENSG00000163491.12	3.20	1.39E-03	NEK10	NIMA-related kinase 10 [Source:HGNC Symbol;Acc:18592]
ENSG00000100726.10	3.42	6.29E-04	TELO2	TEL2, telomere maintenance 2, homolog (S. cerevisiae) [Source:HGNC Symbol;Acc:29099]
ENSG00000072786.8	3.44	5.76E-04	STK10	serine/threonine kinase 10 [Source:HGNC Symbol;Acc:11388]

Table S2: Gene set enrichment for genes identified in the prostate tissue network in Fig.1

GO ID	Gene Ontology Term	Genes	Adjusted p.val (FDR)
GO:0016579	protein deubiquitination	ARRB2,TP53,SHMT2,BRCA1,ESR1,NEDD8,MYC	1.69E-03
GO:0016055	Wnt signaling pathway	MOV10,ARRB2,LRRK2,TNIK,ESR1,APP,CUL3	5.66E-03
GO:2001233	regulation of apoptotic signaling pathway	ARRB2,TP53,LRRK2,BRCA1,YWHAZ,PTTG1IP	9.32E-03
GO:0010821	regulation of mitochondrion organization	ARRB2,TP53,LRRK2,YWHAZ	1.42E-02
GO:0008637	apoptotic mitochondrial changes	ARRB2,TP53,YWHAZ	2.63E-02
GO:0009314	response to radiation	CIRBP,TP53,BRCA1,APP,MYC	2.68E-02

Table S3: Significant genes associated with proctitis in whole blood tissue

Gene (Ensemble ID)	Z-score	P-value	Gene Symbol	Gene symbol definition
Downregulated genes based on Z-scores				
ENSG00000205464.7	-3.62	2.93E-04	ATP6AP1L	ATPase, H+ transporting, lysosomal accessory protein 1-like [Source:HGNC Symbol;Acc:28091]
ENSG00000106077.14	-3.55	3.92E-04	ABHD11	abhydrolase domain containing 11 [Source:HGNC Symbol;Acc:16407]
ENSG00000116786.7	-3.33	8.63E-04	PLEKHM2	pleckstrin homology domain containing, family M (with RUN domain) member 2 [Source:HGNC Symbol;Acc:29131]
ENSG00000197566.5	-3.13	1.74E-03	ZNF624	zinc finger protein 624 [Source:HGNC Symbol;Acc:29254]
ENSG00000008513.10	-3.11	1.88E-03	ST3GAL1	ST3 beta-galactoside alpha-2,3-sialyltransferase 1 [Source:HGNC Symbol;Acc:10862]

ENSG00000171766.11	-3.11	1.89E-03	GATM	glycine amidinotransferase (L-arginine:glycine amidinotransferase) [Source:HGNC Symbol;Acc:4175]
ENSG00000149679.7	-3.09	1.98E-03	CABLES2	Cdk5 and Abl enzyme substrate 2 [Source:HGNC Symbol;Acc:16143]
ENSG00000137819.9	-2.95	3.17E-03	PAQR5	progestin and adipoQ receptor family member V [Source:HGNC Symbol;Acc:29645]
ENSG00000251287.4	-2.94	3.25E-03	ALG1L2	ALG1, chitobiosyldiphosphodolichol beta-mannosyltransferase-like 2 [Source:HGNC Symbol;Acc:37258]
ENSG00000107798.13	-2.89	3.79E-03	LIPA	lipase A, lysosomal acid, cholesterol esterase [Source:HGNC Symbol;Acc:6617]
ENSG00000166669.9	-2.89	3.91E-03	ATF7IP2	activating transcription factor 7 interacting protein 2 [Source:HGNC Symbol;Acc:20397]
ENSG00000197774.8	-2.84	4.44E-03	EME2	essential meiotic endonuclease 1 homolog 2 (S. pombe) [Source:HGNC Symbol;Acc:27289]
ENSG00000097046.8	-2.83	4.66E-03	CDC7	cell division cycle 7 [Source:HGNC Symbol;Acc:1745]
ENSG00000143643.8	-2.81	5.01E-03	TTC13	tetratricopeptide repeat domain 13 [Source:HGNC Symbol;Acc:26204]
ENSG00000100360.10	-2.80	5.06E-03	IFT27	intraflagellar transport 27 homolog (Chlamydomonas) [Source:HGNC Symbol;Acc:18626]
ENSG00000118690.8	-2.73	6.27E-03	ARMC2	armadillo repeat containing 2 [Source:HGNC Symbol;Acc:23045]
ENSG00000211451.7	-2.73	6.37E-03	GNRHR2	gonadotropin-releasing hormone (type 2) receptor 2 [Source:HGNC Symbol;Acc:16341]
ENSG00000106638.11	-2.72	6.47E-03	TBL2	transducin (beta)-like 2 [Source:HGNC Symbol;Acc:11586]
ENSG00000093167.13	-2.71	6.64E-03	LRRFIP2	leucine rich repeat (in FLII) interacting protein 2 [Source:HGNC Symbol;Acc:6703]
ENSG00000104093.9	-2.70	6.83E-03	DMXL2	Dmx-like 2 [Source:HGNC Symbol;Acc:2938]
ENSG00000236056.1	-2.67	7.47E-03	GAPDHP14	glyceraldehyde-3-phosphate dehydrogenase pseudogene 14 [Source:HGNC Symbol;Acc:4160]
ENSG00000166664.9	-2.67	7.59E-03	CHRFAM7A	CHRNA7 (cholinergic receptor, nicotinic, alpha 7, exons 5-10) and FAM7A (family with sequence similarity 7A, exons A-E) fusion [Source:HGNC Symbol;Acc:15781]
ENSG00000170458.9	-2.67	7.69E-03	CD14	CD14 molecule [Source:HGNC Symbol;Acc:1628]
ENSG00000122034.8	-2.65	8.08E-03	GTF3A	general transcription factor IIIA [Source:HGNC Symbol;Acc:4662]
ENSG00000167220.7	-2.57	1.01E-02	HDHD2	haloacid dehalogenase-like hydrolase domain containing 2 [Source:HGNC Symbol;Acc:25364]
ENSG00000142046.10	-2.55	1.08E-02	TMEM91	transmembrane protein 91 [Source:HGNC Symbol;Acc:32393]
ENSG00000178184.11	-2.51	1.20E-02	PARD6G	par-6 partitioning defective 6 homolog gamma (C. elegans) [Source:HGNC Symbol;Acc:16076]
ENSG00000152778.7	-2.50	1.24E-02	IFIT5	interferon-induced protein with tetratricopeptide repeats 5 [Source:HGNC Symbol;Acc:13328]
ENSG00000107745.12	-2.50	1.24E-02	MICU1	mitochondrial calcium uptake 1 [Source:HGNC Symbol;Acc:1530]
ENSG00000215302.4	-2.50	1.25E-02	CTD-3092A11.1	NA
ENSG00000205810.4	-2.48	1.31E-02	KLRC3	killer cell lectin-like receptor subfamily C, member 3 [Source:HGNC Symbol;Acc:6376]
ENSG00000150764.9	-2.46	1.38E-02	DIXDC1	DIX domain containing 1 [Source:HGNC Symbol;Acc:23695]
ENSG00000149503.8	-2.46	1.38E-02	INCENP	inner centromere protein antigens 135/155kDa [Source:HGNC Symbol;Acc:6058]
ENSG00000183955.8	-2.46	1.40E-02	SETD8	SET domain containing (lysine methyltransferase) 8 [Source:HGNC Symbol;Acc:29489]

ENSG00000196189.8	-2.45	1.41E-02	SEMA4A	sema domain, immunoglobulin domain (Ig), transmembrane domain (TM) and short cytoplasmic domain, (semaphorin) 4A [Source:HGNC Symbol;Acc:10729]
ENSG00000163684.7	-2.45	1.43E-02	RPP14	ribonuclease P/MRP 14kDa subunit [Source:HGNC Symbol;Acc:30327]
ENSG00000134884.9	-2.44	1.45E-02	ARGLU1	arginine and glutamate rich 1 [Source:HGNC Symbol;Acc:25482]
ENSG00000217930.3	-2.44	1.45E-02	PAM16	presequence translocase-associated motor 16 homolog (S. cerevisiae) [Source:HGNC Symbol;Acc:29679]
ENSG00000111325.12	-2.43	1.49E-02	OGFOD2	2-oxoglutarate and iron-dependent oxygenase domain containing 2 [Source:HGNC Symbol;Acc:25823]
ENSG00000108107.8	-2.42	1.54E-02	RPL28	ribosomal protein L28 [Source:HGNC Symbol;Acc:10330]
ENSG00000170323.4	-2.42	1.54E-02	FABP4	fatty acid binding protein 4, adipocyte [Source:HGNC Symbol;Acc:3559]
ENSG00000228716.2	-2.41	1.58E-02	DHFR	dihydrofolate reductase [Source:HGNC Symbol;Acc:2861]
ENSG00000227775.3	-2.40	1.63E-02	RP1-283E3.4	NA
ENSG00000114541.10	-2.40	1.65E-02	FRMD4B	FERM domain containing 4B [Source:HGNC Symbol;Acc:24886]
ENSG00000136824.14	-2.39	1.68E-02	SMC2	structural maintenance of chromosomes 2 [Source:HGNC Symbol;Acc:14011]
ENSG00000000457.9	-2.37	1.76E-02	SCYL3	SCY1-like 3 (S. cerevisiae) [Source:HGNC Symbol;Acc:19285]
ENSG00000242588.2	-2.35	1.87E-02	RP11-274B21.1	NA
ENSG00000141086.13	-2.35	1.88E-02	CTRL	chymotrypsin-like [Source:HGNC Symbol;Acc:2524]
ENSG00000127561.10	-2.35	1.89E-02	SYNGR3	synaptogyrin 3 [Source:HGNC Symbol;Acc:11501]
Upregulated genes based on Z-scores				
ENSG00000204516.5	2.33	1.99E-02	MICB	MHC class I polypeptide-related sequence B [Source:HGNC Symbol;Acc:7091]
ENSG00000239736.2	2.33	1.99E-02	CEACAMP3	carcinoembryonic antigen-related cell adhesion molecule pseudogene 3 [Source:HGNC Symbol;Acc:1825]
ENSG00000175538.6	2.35	1.90E-02	KCNE3	potassium voltage-gated channel, Isk-related family, member 3 [Source:HGNC Symbol;Acc:6243]
ENSG00000155542.7	2.35	1.89E-02	SETD9	SET domain containing 9 [Source:HGNC Symbol;Acc:28508]
ENSG00000104731.9	2.35	1.86E-02	KLHDC4	kelch domain containing 4 [Source:HGNC Symbol;Acc:25272]
ENSG00000244733.4	2.36	1.85E-02	RP11-506M13.3	NA
ENSG00000251584.1	2.36	1.85E-02	RP11-440I14.3	NA
ENSG00000198961.5	2.36	1.84E-02	PJA2	praja ring finger 2, E3 ubiquitin protein ligase [Source:HGNC Symbol;Acc:17481]
ENSG00000233297.4	2.36	1.82E-02	RASA4DP	RAS p21 protein activator 4CD, pseudogene [Source:HGNC Symbol;Acc:44226]
ENSG00000184350.8	2.37	1.77E-02	MRGPRE	MAS-related GPR, member E [Source:HGNC Symbol;Acc:30694]
ENSG00000173559.8	2.37	1.76E-02	NABP1	nucleic acid binding protein 1 [Source:HGNC Symbol;Acc:26232]
ENSG00000136250.7	2.38	1.74E-02	AOAH	acyloxyacyl hydrolase (neutrophil) [Source:HGNC Symbol;Acc:548]
ENSG00000078114.14	2.39	1.70E-02	NEBL	nebulette [Source:HGNC Symbol;Acc:16932]
ENSG00000076248.6	2.40	1.66E-02	UNG	uracil-DNA glycosylase [Source:HGNC Symbol;Acc:12572]
ENSG00000272787.1	2.41	1.58E-02	NA	NA

ENSG00000154511.7	2.43	1.52E-02	FAM69A	family with sequence similarity 69, member A [Source:HGNC Symbol;Acc:32213]
ENSG00000186283.9	2.43	1.51E-02	TOR3A	torsin family 3, member A [Source:HGNC Symbol;Acc:11997]
ENSG00000260807.2	2.44	1.46E-02	RP11-161M6.2	NA
ENSG00000184752.8	2.46	1.39E-02	NDUFA12	NADH dehydrogenase (ubiquinone) 1 alpha subcomplex, 12 [Source:HGNC Symbol;Acc:23987]
ENSG00000151470.8	2.47	1.36E-02	C4orf33	chromosome 4 open reading frame 33 [Source:HGNC Symbol;Acc:27025]
ENSG00000143036.12	2.48	1.30E-02	SLC44A3	solute carrier family 44, member 3 [Source:HGNC Symbol;Acc:28689]
ENSG00000139083.6	2.49	1.27E-02	ETV6	ets variant 6 [Source:HGNC Symbol;Acc:3495]
ENSG00000064666.10	2.50	1.26E-02	CNN2	calponin 2 [Source:HGNC Symbol;Acc:2156]
ENSG00000168090.5	2.50	1.23E-02	COPS6	COP9 signalosome subunit 6 [Source:HGNC Symbol;Acc:21749]
ENSG00000101773.12	2.53	1.14E-02	RBBP8	retinoblastoma binding protein 8 [Source:HGNC Symbol;Acc:9891]
ENSG00000107614.17	2.55	1.09E-02	TRDMT1	tRNA aspartic acid methyltransferase 1 [Source:HGNC Symbol;Acc:2977]
ENSG00000263006.2	2.55	1.08E-02	ROCK1P1	Rho-associated, coiled-coil containing protein kinase 1 pseudogene 1 [Source:HGNC Symbol;Acc:37832]
ENSG00000183506.12	2.55	1.06E-02	PI4KAP2	phosphatidylinositol 4-kinase, catalytic, alpha pseudogene 2 [Source:HGNC Symbol;Acc:33577]
ENSG00000117593.8	2.55	1.06E-02	DARS2	aspartyl-tRNA synthetase 2, mitochondrial [Source:HGNC Symbol;Acc:25538]
ENSG00000108106.9	2.58	9.84E-03	UBE2S	ubiquitin-conjugating enzyme E2S [Source:HGNC Symbol;Acc:17895]
ENSG00000168246.5	2.65	7.98E-03	UBTD2	ubiquitin domain containing 2 [Source:HGNC Symbol;Acc:24463]
ENSG00000110274.10	2.67	7.67E-03	CEP164	centrosomal protein 164kDa [Source:HGNC Symbol;Acc:29182]
ENSG00000164053.13	2.68	7.28E-03	ATRIP	ATR interacting protein [Source:HGNC Symbol;Acc:33499]
ENSG00000150753.7	2.71	6.70E-03	CCT5	chaperonin containing TCP1, subunit 5 (epsilon) [Source:HGNC Symbol;Acc:1618]
ENSG00000141068.9	2.74	6.06E-03	KSR1	kinase suppressor of ras 1 [Source:HGNC Symbol;Acc:6465]
ENSG00000100726.10	2.76	5.86E-03	TELO2	TEL2, telomere maintenance 2, homolog (S. cerevisiae) [Source:HGNC Symbol;Acc:29099]
ENSG00000138468.11	2.83	4.63E-03	SENP7	SUMO1/sentrin specific peptidase 7 [Source:HGNC Symbol;Acc:30402]
ENSG00000168016.9	2.85	4.38E-03	TRANK1	tetratricopeptide repeat and ankyrin repeat containing 1 [Source:HGNC Symbol;Acc:29011]
ENSG00000107679.10	2.89	3.87E-03	PLEKHA1	pleckstrin homology domain containing, family A (phosphoinositide binding specific) member 1 [Source:HGNC Symbol;Acc:14335]
ENSG00000152229.14	2.92	3.50E-03	PSTPIP2	proline-serine-threonine phosphatase interacting protein 2 [Source:HGNC Symbol;Acc:9581]
ENSG00000215908.5	2.94	3.29E-03	CROCCP2	ciliary rootlet coiled-coil, rootletin pseudogene 2 [Source:HGNC Symbol;Acc:28170]
ENSG00000105707.9	3.05	2.32E-03	HPN	hepsin [Source:HGNC Symbol;Acc:5155]
ENSG00000134265.8	3.09	2.02E-03	NAPG	N-ethylmaleimide-sensitive factor attachment protein, gamma [Source:HGNC Symbol;Acc:7642]
ENSG00000148180.12	3.29	9.99E-04	GSN	gelsolin [Source:HGNC Symbol;Acc:4620]
ENSG00000095917.9	3.30	9.66E-04	TPSD1	tryptase delta 1 [Source:HGNC Symbol;Acc:14118]

ENSG00000132604.6	3.42	6.22E-04	TERF2	telomeric repeat binding factor 2 [Source:HGNC Symbol;Acc:11729]
ENSG00000089351.10	3.45	5.61E-04	GRAMD1A	GRAM domain containing 1A [Source:HGNC Symbol;Acc:29305]
ENSG00000105711.6	3.57	3.51E-04	SCN1B	sodium channel, voltage-gated, type I, beta subunit [Source:HGNC Symbol;Acc:10586]
ENSG00000145423.4	3.78	1.55E-04	SFRP2	secreted frizzled-related protein 2 [Source:HGNC Symbol;Acc:10777]

Table S4: Gene set enrichment for genes identified in the whole blood tissue network in Fig.2

GO ID	Gene Ontology Term	Genes	Adjusted p.val (FDR)
GO:0006260	DNA replication	TERF2,EGFR,CDC7,BRCA1,ATRIP,RBBP8,SLX4,ORC1,ORC6,RAD50,CDK2,MCM2,DTL,RPA1,RPA2,RPA3	1.12E-10
GO:0031570	DNA integrity checkpoint	FBXO6,BRCA1,CDC5L,ATRIP,MDM2,ORC1,FZR1,CDK2,TP53,DTL,RPA2	6.32E-08
GO:0006289	nucleotide-excision repair	COPS6,RBBP8,DDB1,UBC,SLX4,TP53,RPA1,RPA2,RPA3	4.27E-07
GO:0042769	DNA damage response, detection of DNA damage	DDB1,UBC,DTL,RPA1,RPA2,RPA3	3.25E-06
GO:0000725	recombinational repair	CDC7,BRCA1,RBBP8,SLX4,RAD50,RPA1,RPA2,RPA3	7.81E-06
GO:0000723	telomere maintenance	TERF2,CCT5,SLX4,RAD50,TELO2,RPA1,RPA2,RPA3	3.52E-05
GO:0035825	homologous recombination	SLX4,RAD50,RPA2	2.47E-02

Table S5: Gene set enrichment analysis of overlapping genes with an external cohort (Fig 3)

Category	ID	Term	Genes	adj_pval
Reactome	R-HSA-5617833 Cilium Assembly	2/187	CCT5,IFT27	0.026673704
Reactome	R-HSA-73894 DNA Repair	2/290	RBBP8,UNG	0.026673704
Reactome	R-HSA-1852241 Organelle biogenesis and maintenance	2/263	CCT5,IFT27	0.026673704
MSigDB	PUJANA CHEK2 PCC NETWORK	4/744	UNG,RBBP8,DHFR,CCT5	0.010222045
MSigDB	RASHI RESPONSE TO IONIZING RADIATION 5	2/141	SEMA4A,KSR1	0.015465717
MSigDB	MARTINEZ TP53 TARGETS DN	3/560	ATF7IP2,RBBP8,FRMD4B	0.015465717
GO:MF	Damaged DNA binding	2/74	RBBP8,UNG	0.025039468

Table S6: Comparison of gene expression direction for the 27 overlapping genes

Gene	Name	Direction of expression [External Cohort]	Direction of expression [Whole blood]	Comparison of Direction of Expression
FRMD4B	FERM domain containing 4B	Down	Down	Concordant
UNG	uracil DNA glycosylase	Up	Up	Concordant
RBBP8	RB binding protein 8, endonuclease	Up	Up	Concordant
ST3GAL1	ST3 beta-galactoside alpha-2,3-sialyltransferase 1	Down	Down	Concordant
TRDMT1	tRNA aspartic acid methyltransferase 1	Up	Up	Concordant
ATF7IP2	activating transcription factor 7 interacting protein 2	Down	Down	Concordant
CCT5	chaperonin containing TCP1 subunit 5	Up	Up	Concordant
SEMA4A	semaphorin 4A	Down	Down	Concordant
KSR1	kinase suppressor of ras 1	Up	Up	Concordant
IFT27	intraflagellar transport 27	Down	Down	Concordant
DHFR	dihydrofolate reductase	Down	Down	Concordant

TMEM91	transmembrane protein 91	Down	Down	Concordant
HDHD2	haloacid dehalogenase like hydrolase domain containing 2	Down	Down	Concordant
PARD6G	par-6 family cell polarity regulator gamma	Down	Down	Concordant
GSN	gelsolin	Down	Up	Discordant
PSTPIP2	proline-serine-threonine phosphatase interacting protein 2	Down	Up	Discordant
TRANK1	tetratricopeptide repeat and ankyrin repeat containing 1	Down	Up	Discordant
ABHD11	abhydrolase domain containing 11	Up	Down	Discordant
C4orf33	chromosome 4 open reading frame 33	Down	Up	Discordant
CNN2	calponin 2	Down	Up	Discordant
SMC2	structural maintenance of chromosomes 2	Up	Down	Discordant
TBL2	transducin (beta)-like 2	Up	Down	Discordant
SENP7	SUMO1/sentrin specific peptidase 7	Down	Up	Discordant
CTRL	chymotrypsin like	Up	Down	Discordant
GTF3A	general transcription factor IIIA	Up	Down	Discordant
TTC13	tetratricopeptide repeat domain 13	Up	Down	Discordant
ARGLU1	arginine and glutamate rich 1	Up	Down	Discordant

Table S7: Significant copy number variation associated with proctitis

CNVR ID	Chr	Start	End	Size	Description	z value	p value	Odds Ratio	Lower CI	Upper CI	FDR
CNVR_2810_3	4	3900840	4206068	305229	mixed	5.239295	1.61E-07	7.045454545	3.393982117	14.62542	0.000721
CNVR_2535_2	3	129690192	129896364	206173	mixed	4.491091	7.09E-06	4.908854156	2.451449101	9.829635	0.012584
CNVR_7565_1	11	71293875	71600786	306912	mixed	4.453558	8.45E-06	5.115384615	2.494035738	10.49189	0.012584
CNVR_7646_1	11	89487937	89909274	421338	mixed	4.19543	2.72E-05	4.984126984	2.353384475	10.55566	0.030436
CNVR_505_1	1	121343784	144546934	23203151	mixed	4.103306	4.07E-05	4.210526316	2.118915294	8.366796	0.036412
CNVR_9133_2	15	25392077	25506567	114491	mixed	4.027933	5.62E-05	4.0625	2.053777422	8.035879	0.041921
CNVR_7863_1	12	8329500	8498267	168768	mixed	3.990438	6.59E-05	4.904214511	2.245843909	10.70926	0.042115

Table S8: Gene annotation of significant CNV regions from UCSC Browser (GRCh37/hg19)

Chromosome	Strand	Transcription Start Position	Transcription Stop Position	Gene Symbol	Gene Symbol Description
chr4	+	3941972	3941998	DQ584669	Homo sapiens piRNA piR-51781, complete sequence.
chr4	-	3943668	3957148	FAM86EP	Homo sapiens family with sequence similarity 86, member E, pseudogene (FAM86EP), non-coding RNA.
chr4	-	3943668	3957148	FAM86EP	Homo sapiens family with sequence similarity 86, member E, pseudogene (FAM86EP), non-coding RNA.
chr4	+	4034896	4076783	BC042823	Homo sapiens cDNA clone IMAGE:5275587.
chr4	-	4190529	4228621	OTOP1	Homo sapiens otopetrin 1 (OTOP1), mRNA.
chr3	+	129693235	129696781	TRH	Homo sapiens thyrotropin-releasing hormone (TRH), mRNA.
chr3	+	129800673	129817233	ALG1L2	Homo sapiens ALG1, chitobiosyldiphosphodolichol beta-mannosyltransferase-like 2 (ALG1L2), mRNA.
chr3	+	129800673	129817233	ALG1L2	Homo sapiens ALG1, chitobiosyldiphosphodolichol beta-mannosyltransferase-like 2 (ALG1L2), mRNA.
chr3	-	129816624	129822720	FAM86HP	Homo sapiens family with sequence similarity 86, member H, pseudogene (FAM86HP), non-coding RNA.
chr3	-	129816624	129830276	FAM86HP	Homo sapiens family with sequence similarity 86, member H, pseudogene (FAM86HP), non-coding RNA.
chr11	-	71292900	71293921	KRTAP5-11	Homo sapiens keratin associated protein 5-11 (KRTAP5-11), mRNA.
chr11	+	71498556	71512280	FAM86C1	Homo sapiens family with sequence similarity 86, member C1 (FAM86C1), transcript variant 1, mRNA.
chr11	+	71498556	71512280	FAM86C1	Homo sapiens family with sequence similarity 86, member C1 (FAM86C1), transcript variant 3, mRNA.
chr11	+	71498556	71512280	FAM86C1	Homo sapiens family with sequence similarity 86, member C1 (FAM86C1), transcript variant 2, mRNA.
chr11	+	71498556	71512280	FAM86C1	Homo sapiens family with sequence similarity 86, member C1 (FAM86C1), transcript variant 3, mRNA.
chr11	+	71498556	71512280	FAM86C1	Homo sapiens family with sequence similarity 86, member C1 (FAM86C1), transcript variant 2, mRNA.
chr11	-	71505408	71524905	ALG1L9P	Homo sapiens asparagine-linked glycosylation 1-like 9, pseudogene (ALG1L9P), transcript variant 3, non-coding RNA.
chr11	-	71506860	71524905	ALG1L9P	Homo sapiens asparagine-linked glycosylation 1-like 9, pseudogene (ALG1L9P), transcript variant 1, non-coding RNA.
chr11	-	71521023	71524905	ALG1L9P	Homo sapiens asparagine-linked glycosylation 1-like 9, pseudogene (ALG1L9P), transcript variant 2, non-coding RNA.
chr11	+	71544245	71548608	DEFB108B	Homo sapiens defensin, beta 108B (DEFB108B), mRNA.
chr11	-	71576554	71639493	LOC100133315	Homo sapiens transient receptor potential cation channel, subfamily C, member 2-like (LOC100133315), non-coding RNA.
chr11	+	71589498	71595607	LOC100129216	Homo sapiens beta-defensin 131-like (LOC100129216), mRNA.
chr11	-	89530822	89541743	TRIM49	Homo sapiens tripartite motif containing 49 (TRIM49), mRNA.
chr11	+	89553492	89559667	LOC642414	Homo sapiens clone E1 LOC642414 pseudogene mRNA, partial sequence.
chr11	+	89575164	89584136	TRIM53AP	Homo sapiens tripartite motif containing 53A, pseudogene (TRIM53AP), non-coding RNA.
chr11	-	89603605	89609185	TRIM64B	Homo sapiens tripartite motif containing 64B (TRIM64B), mRNA.
chr11	-	89644578	89653576	TRIM49D2P	Homo sapiens tripartite motif containing 49D2, pseudogene (TRIM49D2P), mRNA.

chr11	+	89657231	89666229	TRIM49D2P	Homo sapiens tripartite motif containing 49D2, pseudogene (TRIM49D2P), mRNA.
chr11	+	89673687	89673722	MIR5692A1	Homo sapiens microRNA 5692a-1 (MIR5692A1), microRNA.
chr11	+	89701671	89707240	TRIM64	Homo sapiens tripartite motif containing 64 (TRIM64), mRNA.
chr11	-	89726708	89735676	TRIM53AP	Homo sapiens tripartite motif containing 53A, pseudogene (TRIM53AP), non-coding RNA.
chr11	-	89751201	89757434	LOC642414	Homo sapiens clone E1 LOC642414 pseudogene mRNA, partial sequence.
chr11	+	89764273	89775193	TRIM49C	Homo sapiens tripartite motif containing 49C (TRIM49C), mRNA.
chr11	-	89794201	89796159	LOC440061	Homo sapiens unknown mRNA sequence.
chr11	+	89819117	89820299	UBTFL1	Homo sapiens upstream binding transcription factor, RNA polymerase I-like 1 (UBTFL1), mRNA.
chr11	+	89867817	89892505	NAALAD2	Homo sapiens N-acetylated alpha-linked acidic dipeptidase 2 (NAALAD2), mRNA.
chr11	+	89867817	89897369	NAALAD2	Homo sapiens N-acetylated alpha-linked acidic dipeptidase 2 (NAALAD2), mRNA.
chr11	+	89867817	89925779	NAALAD2	Homo sapiens N-acetylated alpha-linked acidic dipeptidase 2 (NAALAD2), mRNA.
chr11	+	89867817	89925779	NAALAD2	Homo sapiens N-acetylated alpha-linked acidic dipeptidase 2 (NAALAD2), mRNA.
chr11	+	89867817	89925779	NAALAD2	Homo sapiens N-acetylated alpha-linked acidic dipeptidase 2 (NAALAD2), mRNA.
chr1	+	142618798	143257763	CR936796	Homo sapiens PNAS-130 mRNA, complete cds.
chr1	+	142660105	142660135	DQ579288	Homo sapiens piRNA piR-47400, complete sequence.
chr1	-	142672190	142672219	DQ586768	Homo sapiens piRNA piR-53880, complete sequence.
chr1	+	142688238	142688268	DQ579288	Homo sapiens piRNA piR-47400, complete sequence.
chr1	-	142689205	142689235	DQ583161	Homo sapiens piRNA piR-50272, complete sequence.
chr1	+	142689523	142689553	DQ590589	Homo sapiens piRNA piR-32372, complete sequence.
chr1	-	142697420	142713605	ANKRD20A12P	Homo sapiens ankyrin repeat domain 20 family, member A12, pseudogene (ANKRD20A12P), non-coding RNA.
chr1	-	142803223	142888797	BC053679	Homo sapiens cDNA: FLJ22715 fis, clone HSI13726.
chr1	+	142803530	142826645	BC071797	Homo sapiens, clone IMAGE:4720764, mRNA.
chr1	-	142804898	142890670	BC029473	Homo sapiens cDNA clone IMAGE:4723680, **** WARNING: chimeric clone ****.
chr1	-	142851394	142851424	DQ590126	Homo sapiens piRNA piR-47400, complete sequence.
chr1	+	142853227	142855999	DQ592442	Homo sapiens cDNA FLJ35140 fis, clone PLACE6009524.
chr1	+	143119060	143163748	AK056396	Homo sapiens PNAS-130 mRNA, complete cds.
chr1	-	143168647	143168675	DQ586768	Homo sapiens piRNA piR-53880, complete sequence.
chr1	+	143184707	143184737	DQ579288	Homo sapiens piRNA piR-47400, complete sequence.
chr1	+	143185980	143186010	DQ590589	Homo sapiens piRNA piR-32372, complete sequence.
chr1	-	143286955	143286985	DQ587539	Homo sapiens piRNA piR-32372, complete sequence.
chr1	-	143289062	143289092	DQ590126	Homo sapiens piRNA piR-47400, complete sequence.
chr1	-	143402283	143402313	DQ587539	Homo sapiens piRNA piR-32372, complete sequence.
chr1	-	143403553	143403583	DQ590126	Homo sapiens piRNA piR-47400, complete sequence.
chr1	+	143419286	143419314	DQ596206	Homo sapiens piRNA piR-53880, complete sequence.

chr1	-	143424332	143467651	BC070106	Homo sapiens cDNA clone IMAGE:30343207.
chr1	-	143431367	143431397	DQ590126	Homo sapiens piRNA piR-47400, complete sequence.
chr1	-	143647638	143744587	LINC00875	Homo sapiens long intergenic non-protein coding RNA 875 (LINC00875), non-coding RNA.
chr1	+	143687129	143705973	LOC100130000	Homo sapiens phosphodiesterase 4D interacting protein pseudogene (LOC100130000), non-coding RNA.
chr1	+	143687129	143714180	LOC100130000	Homo sapiens phosphodiesterase 4D interacting protein pseudogene (LOC100130000), non-coding RNA.
chr1	+	143690027	143690101	TRNA_Asn	transfer RNA Asn (anticodon GTT)
chr1	+	143702303	143702331	DQ571491	Homo sapiens piRNA piR-31603, complete sequence.
chr1	-	143717587	143744587	LINC00875	Homo sapiens long intergenic non-protein coding RNA 875 (LINC00875), non-coding RNA.
chr1	-	143718512	143744587	LINC00875	Homo sapiens long intergenic non-protein coding RNA 875 (LINC00875), non-coding RNA.
chr1	-	143719238	143744587	LINC00875	Homo sapiens long intergenic non-protein coding RNA 875 (LINC00875), non-coding RNA.
chr1	-	143767143	143767881	PPIAL4G	Homo sapiens peptidylprolyl isomerase A (cyclophilin A)-like 4G (PPIAL4G), mRNA.
chr1	-	143879831	143879905	TRNA_Asn	transfer RNA Asn (anticodon GTT)
chr1	-	143896451	143913143	FAM72D	Homo sapiens family with sequence similarity 72, member D (FAM72D), mRNA.
chr1	-	143896451	143913143	FAM72D	Homo sapiens family with sequence similarity 72, member D (FAM72D), mRNA.
chr1	+	143915747	144094477	SRGAP2B	Homo sapiens SLIT-ROBO Rho GTPase activating protein 2B (SRGAP2B), mRNA.
chr1	+	144146810	144830407	NBPF8	Homo sapiens neuroblastoma breakpoint family, member 8 (NBPF8), transcript variant 3, non-coding RNA.
chr1	+	144146810	146467744	LOC100288142	Homo sapiens neuroblastoma breakpoint family member (LOC100288142), mRNA.
chr1	+	144146810	144830407	NBPF9	Homo sapiens neuroblastoma breakpoint family, member 9 (NBPF9), transcript variant 2, mRNA.
chr1	+	144148789	144830407	NBPF8	Homo sapiens neuroblastoma breakpoint family, member 8 (NBPF8), transcript variant 3, non-coding RNA.
chr1	+	144150981	144167711	LOC100288142	Homo sapiens neuroblastoma breakpoint family member (LOC100288142), mRNA.
chr1	+	144151518	144830407	LOC100288142	Homo sapiens neuroblastoma breakpoint family member (LOC100288142), mRNA.
chr1	+	144160410	144201052	LOC100288142	Homo sapiens neuroblastoma breakpoint family member (LOC100288142), mRNA.
chr1	+	144162000	144167711	LOC100288142	Homo sapiens neuroblastoma breakpoint family member (LOC100288142), mRNA.
chr1	+	144162000	144186823	LOC100288142	Homo sapiens neuroblastoma breakpoint family member (LOC100288142), mRNA.
chr1	+	144162887	144182059	LOC100288142	Homo sapiens neuroblastoma breakpoint family member (LOC100288142), mRNA.
chr1	+	144171303	144172451	AF420437	Homo sapiens AB13 precursor RNA, partial cds.
chr1	+	144176438	144830407	NBPF8	Homo sapiens neuroblastoma breakpoint family, member 8 (NBPF8), transcript variant 3, non-coding RNA.
chr1	+	144179473	144223374	LOC100288142	Homo sapiens neuroblastoma breakpoint family member (LOC100288142), mRNA.
chr1	+	144181950	144186823	LOC100288142	Homo sapiens neuroblastoma breakpoint family member (LOC100288142), mRNA.

chr1	+	144183587	144830407	NBPF8	Homo sapiens neuroblastoma breakpoint family, member 8 (NBPF8), transcript variant 3, non-coding RNA.
chr1	+	144184251	145339512	LOC100288142	Homo sapiens neuroblastoma breakpoint family member (LOC100288142), mRNA.
chr1	+	144185827	144223374	LOC100288142	Homo sapiens neuroblastoma breakpoint family member (LOC100288142), mRNA.
chr1	+	144189011	144827928	LOC100288142	Homo sapiens neuroblastoma breakpoint family member (LOC100288142), mRNA.
chr1	+	144190581	144830407	NBPF8	Homo sapiens neuroblastoma breakpoint family, member 8 (NBPF8), transcript variant 3, non-coding RNA.
chr1	+	144190581	144209025	LOC100288142	Homo sapiens neuroblastoma breakpoint family member (LOC100288142), mRNA.
chr1	+	144196230	144201052	LOC100288142	Homo sapiens neuroblastoma breakpoint family member (LOC100288142), mRNA.
chr1	+	144196230	144209025	LOC100288142	Homo sapiens neuroblastoma breakpoint family member (LOC100288142), mRNA.
chr1	+	144209457	144212180	AL050141	Homo sapiens mRNA; cDNA DKFZp586O031 (from clone DKFZp586O031).
chr1	+	144218498	144830407	NBPF8	Homo sapiens neuroblastoma breakpoint family, member 8 (NBPF8), transcript variant 3, non-coding RNA.
chr1	-	144275887	144290006	AX746564	Homo sapiens cDNA FLJ33341 fis, clone BRACE2002582.
chr1	-	144300511	144340773	LINC00623	Homo sapiens long intergenic non-protein coding RNA 623 (LINC00623), non-coding RNA.
chr1	-	144300511	144340773	LINC00623	Homo sapiens long intergenic non-protein coding RNA 623 (LINC00623), non-coding RNA.
chr1	-	144300511	144341755	LOC728875	Homo sapiens uncharacterized LOC728875 (LOC728875), non-coding RNA.
chr1	+	144301610	144301684	TRNA_Asn	transfer RNA Asn (anticodon GTT)
chr1	-	144308613	144308687	TRNA_Asn	transfer RNA Asn (anticodon GTT)
chr1	-	144339562	144340773	LINC00623	Homo sapiens long intergenic non-protein coding RNA 623 (LINC00623), non-coding RNA.
chr1	-	144340773	144341077	BC047032	Homo sapiens cDNA FLJ33341 fis, clone BRACE2002582.
chr1	-	144363461	144364246	PPIAL4B	Homo sapiens peptidylprolyl isomerase A (cyclophilin A)-like 4B (PPIAL4B), mRNA.
chr1	-	144456162	144470244	AX746564	Homo sapiens cDNA FLJ33341 fis, clone BRACE2002582.
chr1	-	144480744	144521009	LOC728875	Homo sapiens uncharacterized LOC728875 (LOC728875), non-coding RNA.
chr1	-	144480745	144521969	LOC728875	Homo sapiens uncharacterized LOC728875 (LOC728875), non-coding RNA.
chr1	-	144480745	144521009	LOC728875	Homo sapiens uncharacterized LOC728875 (LOC728875), non-coding RNA.
chr1	+	144481839	144481913	TRNA_Asn	transfer RNA Asn (anticodon GTT)
chr1	-	144488842	144488916	TRNA_Asn	transfer RNA Asn (anticodon GTT)
chr1	-	144514872	144519720	AK094156	Homo sapiens primary neuroblastoma cDNA, clone:Nbla04072, full insert sequence.
chr15	+	25362556	25420017	IPW	Homo sapiens imprinted in Prader-Willi syndrome (non-protein coding) (IPW), non-coding RNA.
chr15	+	25415869	25415951	SNORD115-1	Homo sapiens small nucleolar RNA, C/D box 115-1 (SNORD115-1), small nucleolar RNA.
chr15	+	25417781	25417863	SNORD115-2	Homo sapiens small nucleolar RNA, C/D box 115-2 (SNORD115-2), small nucleolar RNA.

chr15	+	25418125	25427252	SNURF-SNRPN	Homo sapiens clone Rt-5 SNURF-SNRPN mRNA, downstream untranslated exons, alternatively spliced.
chr15	+	25420073	25420155	SNORD115-3	Homo sapiens small nucleolar RNA, C/D box 115-3 (SNORD115-3), small nucleolar RNA.
chr15	+	25421978	25422060	SNORD115-4	Homo sapiens small nucleolar RNA, C/D box 115-4 (SNORD115-4), small nucleolar RNA.
chr15	+	25423884	25423966	SNORD115-5	Homo sapiens small nucleolar RNA, C/D box 115-5 (SNORD115-5), small nucleolar RNA.
chr15	+	25425643	25425725	SNORD115-6	Homo sapiens small nucleolar RNA, C/D box 115-6 (SNORD115-6), small nucleolar RNA.
chr15	+	25426956	25430721	SNURF-SNRPN	Homo sapiens clone Rt-7 SNURF-SNRPN mRNA, downstream untranslated exons, alternatively spliced.
chr15	+	25427531	25427613	SNORD115-7	Homo sapiens small nucleolar RNA, C/D box 115-7 (SNORD115-7), small nucleolar RNA.
chr15	+	25427858	25431154	SNURF-SNRPN	Homo sapiens clone Rt-9 SNURF-SNRPN mRNA, downstream untranslated exons, alternatively spliced.
chr15	+	25429452	25429534	SNORD115-8	Homo sapiens small nucleolar RNA, C/D box 115-8 (SNORD115-8), small nucleolar RNA.
chr15	+	25430777	25430859	SNORD115-9	Homo sapiens small nucleolar RNA, C/D box 115-9 (SNORD115-9), small nucleolar RNA.
chr15	+	25432682	25432763	SNORD115-10	Homo sapiens small nucleolar RNA, C/D box 115-10 (SNORD115-10), small nucleolar RNA.
chr15	+	25434560	25434642	SNORD115-11	Homo sapiens small nucleolar RNA, C/D box 115-11 (SNORD115-11), small nucleolar RNA.
chr15	+	25436386	25442649	SNURF-SNRPN	Homo sapiens clone Rt-11 SNURF-SNRPN mRNA, downstream untranslated exons, alternatively spliced.
chr15	+	25436562	25436644	SNORD115-9	Homo sapiens small nucleolar RNA, C/D box 115-9 (SNORD115-9), small nucleolar RNA.
chr15	+	25438467	25438549	SNORD115-13	Homo sapiens small nucleolar RNA, C/D box 115-13 (SNORD115-13), small nucleolar RNA.
chr15	+	25440067	25440148	SNORD115-14	Homo sapiens small nucleolar RNA, C/D box 115-14 (SNORD115-14), small nucleolar RNA.
chr15	+	25442574	25459165	SNURF-SNRPN	Homo sapiens clone Rt-13I SNURF-SNRPN mRNA, downstream untranslated exons, alternatively spliced.
chr15	+	25442574	25459165	SNURF-SNRPN	Homo sapiens clone Rt-13I SNURF-SNRPN mRNA, downstream untranslated exons, alternatively spliced.
chr15	+	25444594	25444676	SNORD115-16	Homo sapiens small nucleolar RNA, C/D box 115-16 (SNORD115-16), small nucleolar RNA.
chr15	+	25446469	25446551	SNORD115-17	Homo sapiens small nucleolar RNA, C/D box 115-17 (SNORD115-17), small nucleolar RNA.
chr15	+	25448373	25448455	SNORD115-17	Homo sapiens small nucleolar RNA, C/D box 115-17 (SNORD115-17), small nucleolar RNA.
chr15	+	25449503	25449585	SNORD115-17	Homo sapiens small nucleolar RNA, C/D box 115-17 (SNORD115-17), small nucleolar RNA.
chr15	+	25451408	25477615	SNORD115-15	Homo sapiens small nucleolar RNA, C/D box 115-15 (SNORD115-15), small nucleolar RNA.
chr15	+	25455064	25455146	SNORD115-22	Homo sapiens small nucleolar RNA, C/D box 115-22 (SNORD115-22), small nucleolar RNA.
chr15	+	25456838	25457180	PAR4	Homo sapiens Prader-Willi/Angelman region gene 4 (PAR4), non-coding RNA.
chr15	+	25458605	25467437	SNURF-SNRPN	Homo sapiens clone Rt-13I SNURF-SNRPN mRNA, downstream untranslated exons, alternatively spliced.
chr15	+	25458805	25458876	SNORD115-24	Homo sapiens small nucleolar RNA, C/D box 115-24 (SNORD115-24), small nucleolar RNA.

chr15	+	25460687	25460769	SNORD115-25	Homo sapiens small nucleolar RNA, C/D box 115-25 (SNORD115-25), small nucleolar RNA.
chr15	+	25463498	25479764	SNURF-SNRPN	Homo sapiens clone Rt-15 SNURF-SNRPN mRNA, downstream untranslated exons, alternatively spliced.
chr15	+	25463763	25463845	SNORD115-26	Homo sapiens small nucleolar RNA, C/D box 115-26 (SNORD115-26), small nucleolar RNA.
chr15	+	25465649	25465725	SNORD115-27	Homo sapiens small nucleolar RNA, C/D box 115-27 (SNORD115-27), small nucleolar RNA.
chr15	+	25467500	25467574	SNORD115-28	Homo sapiens small nucleolar RNA, C/D box 115-28 (SNORD115-28), small nucleolar RNA.
chr15	+	25468392	25468474	SNORD115-11	Homo sapiens small nucleolar RNA, C/D box 115-11 (SNORD115-11), small nucleolar RNA.
chr15	+	25470349	25470431	SNORD115-30	Homo sapiens small nucleolar RNA, C/D box 115-30 (SNORD115-30), small nucleolar RNA.
chr15	+	25472255	25472337	SNORD115-31	Homo sapiens small nucleolar RNA, C/D box 115-31 (SNORD115-31), small nucleolar RNA.
chr15	+	25474113	25474195	SNORD115-32	Homo sapiens small nucleolar RNA, C/D box 115-32 (SNORD115-32), small nucleolar RNA.
chr15	+	25475984	25476066	SNORD115-33	Homo sapiens small nucleolar RNA, C/D box 115-33 (SNORD115-33), small nucleolar RNA.
chr15	+	25479393	25479475	SNORD115-35	Homo sapiens small nucleolar RNA, C/D box 115-35 (SNORD115-35), small nucleolar RNA.
chr15	+	25481231	25481313	SNORD115-11	Homo sapiens small nucleolar RNA, C/D box 115-11 (SNORD115-11), small nucleolar RNA.
chr15	+	25481555	25620623	SNURF-SNRPN	Homo sapiens clone Rt-16 SNURF-SNRPN mRNA, downstream untranslated exons, alternatively spliced.
chr15	+	25483132	25483214	SNORD115-37	Homo sapiens small nucleolar RNA, C/D box 115-37 (SNORD115-37), small nucleolar RNA.
chr15	+	25484984	25485066	SNORD115-38	Homo sapiens small nucleolar RNA, C/D box 115-38 (SNORD115-38), small nucleolar RNA.
chr15	+	25486892	25486974	SNORD115-39	Homo sapiens small nucleolar RNA, C/D box 115-39 (SNORD115-39), small nucleolar RNA.
chr15	+	25488760	25488842	SNORD115-40	Homo sapiens small nucleolar RNA, C/D box 115-40 (SNORD115-40), small nucleolar RNA.
chr15	+	25490624	25490706	SNORD115-41	Homo sapiens small nucleolar RNA, C/D box 115-41 (SNORD115-41), small nucleolar RNA.
chr15	+	25492491	25492573	SNORD115-10	Homo sapiens small nucleolar RNA, C/D box 115-10 (SNORD115-10), small nucleolar RNA.
chr15	+	25494344	25494426	SNORD115-11	Homo sapiens small nucleolar RNA, C/D box 115-11 (SNORD115-11), small nucleolar RNA.
chr15	+	25496005	25496087	SNORD115-44	Homo sapiens small nucleolar RNA, C/D box 115-44 (SNORD115-44), small nucleolar RNA.
chr12	+	8325149	8332642	ZNF705A	Homo sapiens zinc finger protein 705A (ZNF705A), mRNA.
chr12	+	8332804	8353596	FAM66C	Homo sapiens family with sequence similarity 66, member C (FAM66C), non-coding RNA.
chr12	+	8332804	8353596	FAM66C	Homo sapiens family with sequence similarity 66, member C (FAM66C), non-coding RNA.
chr12	+	8332804	8356982	FAM66C	Homo sapiens family with sequence similarity 66, member C (FAM66C), non-coding RNA.
chr12	+	8332804	8368747	FAM66C	Homo sapiens family with sequence similarity 66, member C (FAM66C), non-coding RNA.
chr12	-	8373855	8380214	FAM90A1	Homo sapiens family with sequence similarity 90, member A1 (FAM90A1), mRNA.

chr12	-	8373855	8380214	FAM90A1	Homo sapiens family with sequence similarity 90, member A1 (FAM90A1), mRNA.
chr12	-	8383644	8395542	FAM86FP	Homo sapiens family with sequence similarity 86, member F, pseudogene (FAM86FP), non-coding RNA.

Table S9: Significant functions identified for the network constructed in IPA® (Fig.4) of genes in CNV regions

Categories	Functions	Diseases or Functions Annotation	p-Value	Molecules
Hair and Skin Development and Function	anagen	Anagen	6.97E-04	TRH
Endocrine System Disorders, Hereditary Disorder, Metabolic Disease, Organismal Injury and Abnormalities	thyrotropin-releasing hormone deficiency	Thyrotropin-releasing hormone deficiency	6.97E-04	TRH
Molecular Transport	transmembrane transport	Transmembrane transport of H+	1.66E-02	OTOP1
Cell Death and Survival, Organismal Injury and Abnormalities	apoptosis	Apoptosis of keratinocytes	2.00E-02	TRH
Cancer, Organismal Injury and Abnormalities, Reproductive System Disease	uterine carcinoma	Uterine carcinoma	2.03E-02	KRTAP5-11, FAM72C/FAM72D, OTOP1, FAM90A1, TRIM49/TRIM49C, DEFB108B, TRIM64/TRIM64B
Cancer, Organismal Injury and Abnormalities	adenoma formation	Adenoma	2.53E-02	TRH, NBPF10 (includes others)
Developmental Disorder, Hereditary Disorder, Organismal Injury and Abnormalities	Prader-Willi syndrome	Prader-Willi syndrome	2.55E-02	IPW
Cancer, Endocrine System Disorders, Organismal Injury and Abnormalities, Reproductive System Disease	pituitary gland adenoma	Pituitary gland adenoma	3.23E-02	TRH
Carbohydrate Metabolism, Small Molecule Biochemistry	synthesis	Synthesis of inositol phosphate	3.43E-02	TRH
Amino Acid Metabolism, Small Molecule Biochemistry	synthesis	Synthesis of amino acids	3.70E-02	NAALAD2
Cell-To-Cell Signaling and Interaction, Embryonic Development	response	Response of embryonic cell lines	3.83E-02	TRIM49/TRIM49C
Cell-To-Cell Signaling and Interaction, Hair and Skin Development and Function	response	Response of epithelial cell lines	3.83E-02	TRIM49/TRIM49C
Cell-To-Cell Signaling and Interaction, Renal and Urological System Development and Function	response	Response of kidney cell lines	3.83E-02	TRIM49/TRIM49C
Cellular Development, Cellular Growth and Proliferation, Connective Tissue Development and Function, Hair and Skin Development and Function, Organ Development, Tissue Development	proliferation	Proliferation of keratinocytes	4.30E-02	TRH

Figure S1: Visual summary of methodology

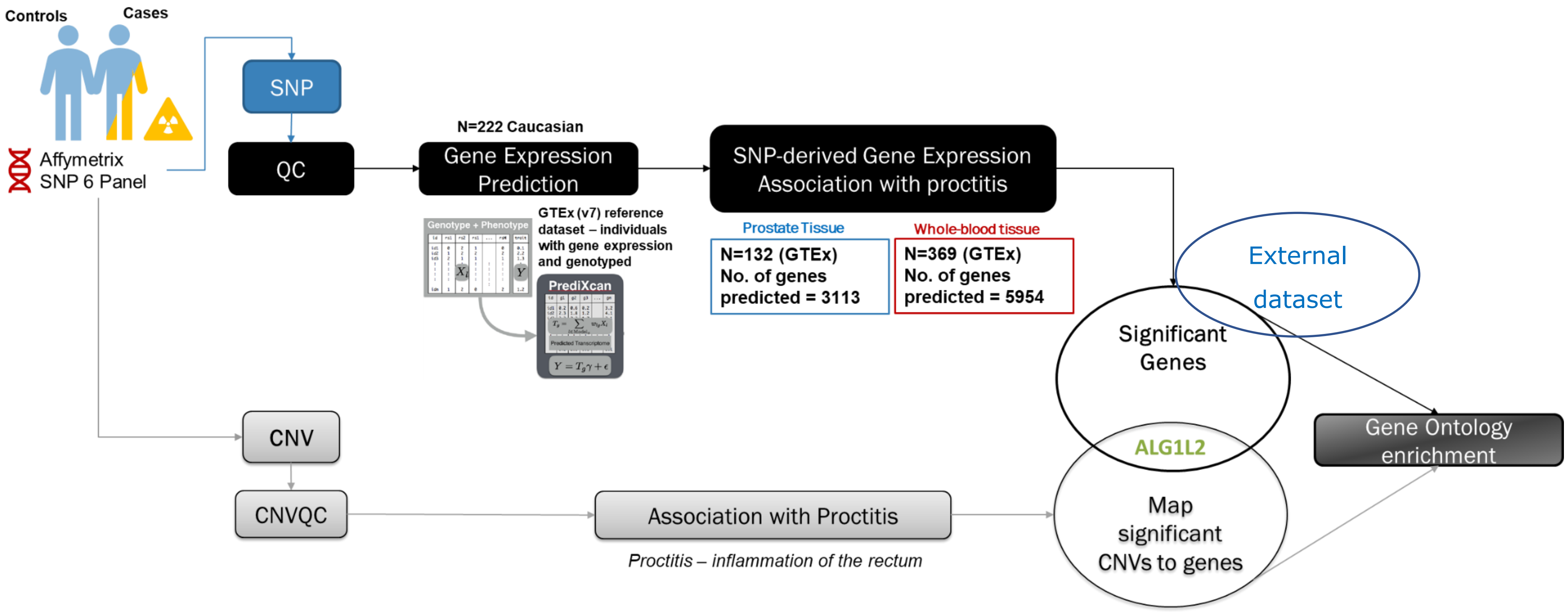

Supplement: Supplementary file 1 — Additional file 1. [file 12885_2020_7457_MOESM1_ESM.pdf]
